# Crystal structure of paddle-wheel sandwich-type [Cu_2_{(CH_3_)_2_CO}{μ-Fe(η^5^-C_5_H_4_C N)_2_}_3_](BF_4_)_2_·(CH_3_)_2_CO

**DOI:** 10.1107/S2056989015001760

**Published:** 2015-01-31

**Authors:** Frank Strehler, Marcus Korb, Heinrich Lang

**Affiliations:** a Technische Universität Chemnitz, Fakultät für Naturwissenschaften, Institut für Chemie, Anorganische Chemie, D-09107 Chemnitz, Germany

**Keywords:** crystal structure, ferrocene-1,1′-dicarbo­nitrile, penta­metallic complex, paddle-wheel, copper(I), coordination chemistry, η^2^,π-inter­action, π–π inter­actions

## Abstract

The mol­ecular structure of (acetone-κ*O*)tris­(μ-ferrocene-1,1′-dicarbo­nitrile-κ^2^ *N*:*N*′)dicopper(I) bis­(tetra­fluorido­borate) acetone monosolvate, [Cu_2_Fe_3_(C_6_H_4_N)_6_(C_3_H_6_O)](BF_4_)_2_·C_3_H_6_O, consists of two Cu^I^ ions bridged by a ferrocene-1,1′-dicarbo­nitrile moiety in a paddle-wheel-architectured sandwich complex with two BF_4_^−^ units as counter-ions. One of the latter is equally disordered over two sets of sites. The two Cu^I^ ions are complexed in a trigonal–planar manner by three nitrile *N*-donor atoms. Further inter­actions by the O atom of an acetone mol­ecule to one of the Cu^I^ atoms and a weak η^2^,π-inter­action of two atoms of a cyclo­penta­dienyl ring to the other Cu^I^ atom complete a distorted trigonal–pyramidal environment for each of the metal ions. A further acetone mol­ecule is also present as a solvent mol­ecule. The crystal packing is consolidated by several π–π inter­actions.

## Abstract 2

The first ferrocenylcarbo­nitrile copper complex is reported. The structure consists of two Cu^I^ ions complexed by ferrocenediyl-1,1′-dicarbo­nitrile forming a paddle-wheel with two acetone mol­ecules, with one coordinating on top of one trigonal–planar-coordinated copper ion, and the other as a packing solvent.

## Chemical context   

The electron-transfer properties of the acetyl­ide function have been investigated intensively by using bridging units of the type —C C—*M*—C C— (*M* = transition metal), showing moderate electron communication between two redox-active metallocenyl termini in the mixed-valence species (see, for example: Lang *et al.*, 2006 ▸; Vives *et al.*, 2006 ▸; Jakob *et al.*, 2009 ▸; Díez *et al.*, 2008 ▸, 2009 ▸; Osella *et al.*, 1998 ▸; Packheiser *et al.*, 2008 ▸; Burgun *et al.*, 2013 ▸). The nitrile group is isoelectronic with the acetyl­ide function; Bonniard *et al.* (2011 ▸) described how an —N C—C_6_H_4_—C N— linkage between two iron fragments prohibits the electronic inter­action between the transition metal atoms, while the isoelectric di(acetyl­ene)–phenyl­ene bridge shows a moderate delocalization. In contrast, a weak electron transfer by generation of the mixed-valence species [Ru(N C*Fc*)(NH_3_)_5_]^3+^ [*Fc* = Fe(η^5^-C_5_H_4_)(η^5^-C_5_H_5_)] has been described (Dowling *et al.*, 1981 ▸). We recently reported on the synthesis, characterization and electrochemical properties of platinum and copper complexes containing a —C N—*M*—N C— (*M* = Cu or Pt) bridging unit between two redox-active ferrocenyl moieties (Strehler *et al.*, 2013 ▸, 2014 ▸) to achieve a direct comparison with the —C C—*M*—C C— building blocks. In addition, the coord­ination of ferrocene-1,1′-dicarbo­nitrile towards PtCl_2_ resulted in an oligomeric complex (Strehler *et al.*, 2014 ▸). In a continuation of this work, we present herein the synthesis and crystal structure of [Cu_2_{(CH_3_)_2_CO}{μ-Fe(η^5^-C_5_H_4_C N)_2_}_3_](BF_4_)_2_·(CH_3_)_2_CO, (I). The synthesis of this compound was realized by comproportionation of elementary copper and a copper(II) salt in the presence of 1,1′-ferrocenediyl dicarbo­nitrile.
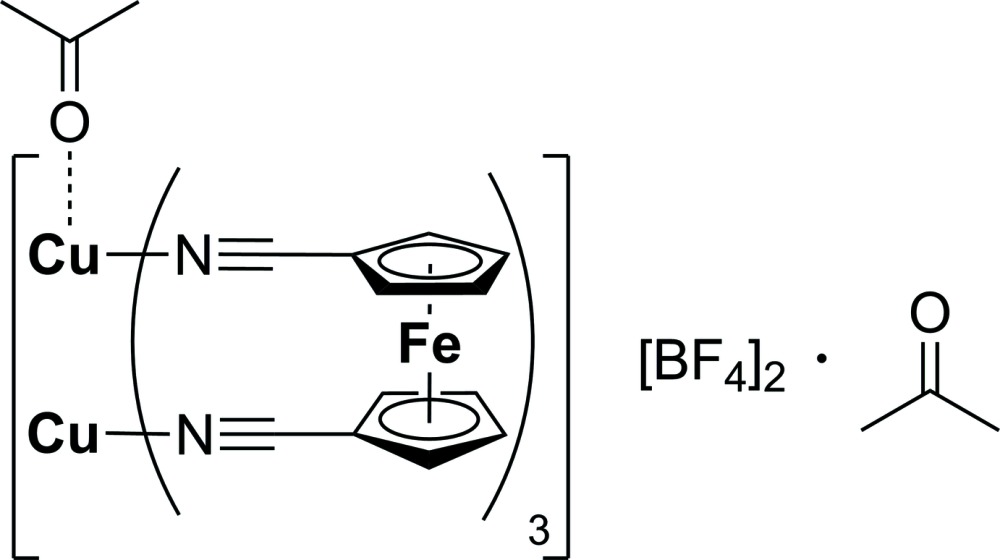


## Structural commentary   

**Figure 1:**
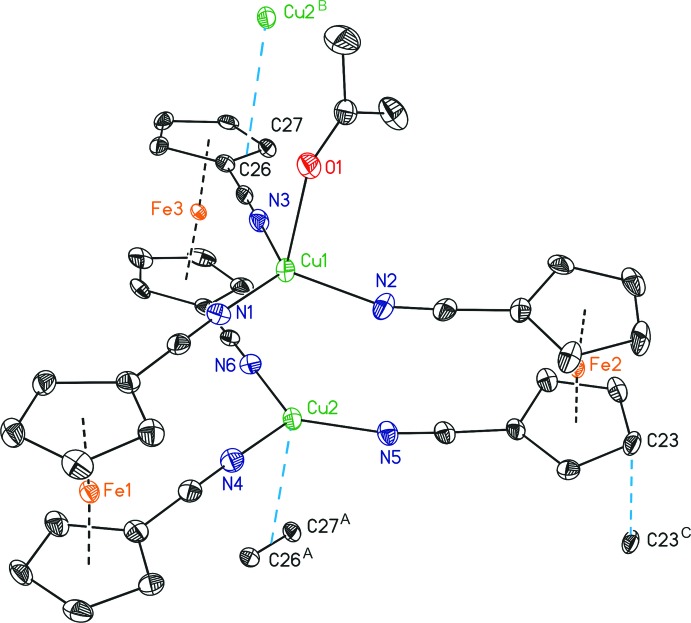
The mol­ecular structure of (I), showing inter­molecular η^2^, π inter­actions between Cu2 and the C26—C27 bond, and short inter­actions between C23 and its symmetry-generated equivalent (Table 1 ▸), with displacement ellipsoids drawn at the 50% probability level. All H atoms, the BF_4_^−^ ions and the non-coordinating acetone solvent mol­ecule have been omitted for clarity. [Symmetry codes: (A) *x* − 1, *y*, *z*; (B) 1 + *x*, *y*, *z*; (C) −*x*, 1 − *y*, 1 − *z*.]

The title compound contains one penta­metallic Cu_2_Fe_3_ complex mol­ecule in the asymmetric unit consisting of two Cu^I^ ions bridged by three 1,1′-ferrocenediyl dicarbo­nitrile ligands that form a triangular paddle-wheel sandwich-type complex with iron⋯iron distances ranging from 9.1739 (13) (Fe2⋯Fe3) to 10.0385 (12) Å (Fe1ctdot;Fe2). The complex crystallizes with two BF_4_^−^ counter-ions and two mol­ecules of acetone. One acetone mol­ecule coordinates with its oxygen atom to Cu1 [Cu1—O1 2.375 (2) Å], leading to an 18 VE complex and an overall distorted trigonal–pyramidal environment. The Cu2 ion exhibits a weak inter­molecular η^2^, π inter­action [3.1520 (6) Å; Table 1 ▸, Fig. 1 ▸] with two atoms of an adjacent cyclo­penta­dienyl ring, and thus, only a 16 VE complex is present. The deviation from the N_3_ plane is increased for Cu1 [0.1883 (16) Å] as compared to Cu2 [0.0602 (16) Å] due to a stronger inter­action with the axial moiety. The Cu⋯Cu distance [3.3818 (7) Å] exceeds the sum of the van der Waals radii (Σ = 2.80 Å; Bondi, 1964 ▸), indicating that the Cu^I^ ions do not inter­act with each other.

**Figure 2:**
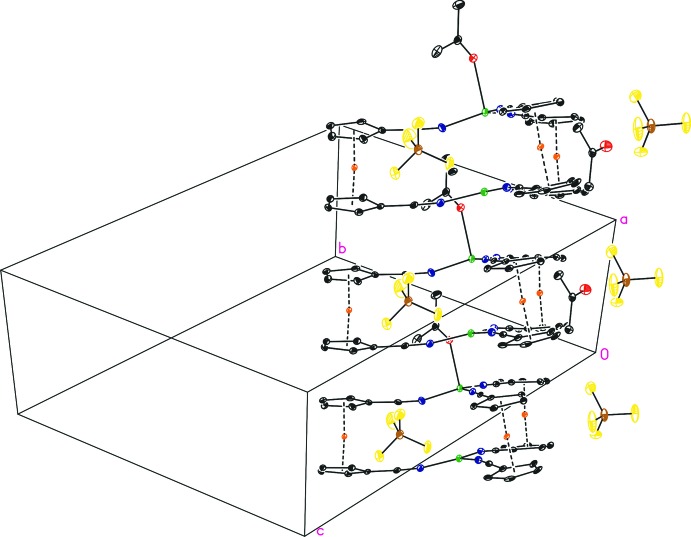
Packing of mol­ecular layers in the crystal structure of (I), with displacement ellipsoids drawn at the 30% probability level. All H atoms have been omitted for clarity. The disorder of one of the counter-anions is not shown.

The two faces of the sandwich-type complex consist of almost coplanar cyclo­penta­dienyl aromatics and central planes formed by three nitro­gen atoms that are also almost coplanar towards the C_5_ planes. However, one cyclo­penta­dienyl ring of each site deviates from coplanarity (Table 2 ▸), which results in a slight bending of the whole complex (Fig. 2 ▸). The ferrocenyl cyclo­penta­dienyl moieties virtually exhibit ecliptic conformations [4.5 (2) to 6.4 (2) °], with synperiplanar-oriented carbo­nitrile substituents towards each other. Maximum deviations from this plane are observed for N5 [0.289 (7) Å] and Cu2 [0.825 (9) Å].

## Supra­molecular features   

Besides the already noted inter­molecular inter­action between Cu2 and the mid-point of the C26—C27 bond, π–π inter­actions are present in the crystal packing between the C23 atom and its symmetry-generated equivalent [3.167 (6) Å; Table 1 ▸]. All other π inter­actions occur almost perpendicular to the involved C_5_ ring [α C_5_⋯C23, 92.2 (2) °; α C_5_⋯Cu2, 93.23 (1) °; Table 1 ▸]. Compound (I) forms a layer-type structure parallel to (11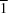) (Fig. 2 ▸), in which the coordinating acetone mol­ecule is part of the overlaying layer. The second acetone mol­ecule is present in each layer and does not exhibit any notable inter­molecular inter­actions. The distances between two layers are in the range of the above-mentioned inter­actions.

## Database survey   

Since the first synthesis of 1,1′-di­cyano­ferrocene (Osgerby & Pauson, 1961 ▸), only one example of a crystal structure has been reported, that of the mol­ecule itself (Altmannshofer *et al.*, 2008 ▸) which exhibits a similar synperiplanar torsion (–2.2°) of the cyclo­penta­dienyl rings to that in (I). Further mol­ecules bearing one nitrilo substituent at the ferrocenyl backbone include a penta­carbonyl tungsten complex with the second nitrilo functionality involved in a 2,3-di­hydro-1,2,3-aza­diphosphete (Helten *et al.*, 2010 ▸) and recently published square-planar *cis*- and *trans*-platinum(II) complexes of cyano­ferrocene (Strehler *et al.*, 2014 ▸).

Trigonal–planar (hetero-bimetallic) Cu^I^ complexes are well described in the literature (Lang *et al.*, 1995 ▸, 2000 ▸, 2006 ▸; Buschbeck *et al.*, 2011 ▸; Ferrara *et al.*, 1987 ▸; Köhler *et al.*, 1998 ▸; Frosch *et al.*, 2000 ▸, 2001 ▸; Janssen *et al.*, 1995 ▸; Spek, 2007 ▸). However, the coordination to a further carbon atom with similar short Cu⋯C distances has rarely been described (Cu⋯C distances are given in parentheses) [Dong *et al.*, 2008 ▸ (3.538 and 3.583 Å); Chesnut *et al.*, 1998 ▸ (3.126 Å); Fu *et al.*, 2008 ▸ (3.577 and 3.561 Å); Benmansour *et al.*, 2009 ▸ (3.088 and 3.519 Å] compared to 3.1520 (6) Å in (I). They mainly contain cyanide mol­ecules acting as donating ligands that are partially replaced by aromatic *N*-donating mol­ecules.

Regarding nitriles as donating mol­ecules, a tris­(benzo­nitrilo)­copper(I) perchlorate complex (Bowmaker *et al.*, 2004 ▸) has been reported, exhibiting a similar trigonal–planar coordination environment including the counter-ion acting as one axial ligand with a similar Cu—O distance of 2.404 (4) Å [compared to 2.375 (2) Å in (I)]. This results in a distorted trigonal-pyramidal environment with N—Cu—N angles slightly more varying [105.4 (2) to 130.4 (2)°] than in (I) [113.63 (11) to 128.97 (11)°], but Cu—N distances [1.906 (4)–1.958 (4) Å] in the same range as in (I) [1.911 (3)–1.960 (3) Å].

## Synthesis and crystallization   

Ferrocene-1,1′-dicarbo­nitrile was prepared according to a published procedure (Strehler *et al.*, 2014 ▸). Synthesis of [Cu_2_{(CH_3_)_2_CO}{μ-Fe(η^5^-C_5_H_4_C N)_2_}_3_](BF_4_)_2_·(CH_3_)_2_CO: Copper powder (6 mg, 0.09 mmol), Cu(BF_4_)_2_·5H_2_O (12.5 mg, 0.05 mmol) and ferrocene-1,1′-dicarbo­nitrile (50.0 mg, 0.20 mmol) were stirred in 5 ml of di­chloro­methane at room temperature overnight. The resulting orange precipitate was filtered off using zeolite and washed several times with 20 ml of di­chloro­methane until the filtrate was colorless. The residue was taken up in acetone and this solution was evaporated to dryness using a rotary evaporator affording (I) as an orange solid. The evaporation was stopped before dryness, small orange crystals of (I) suitable for X-ray crystal structure analysis could be isolated. On further drying, the crystals decomposed due to evaporation of acetone from the crystal. Yield: 42 mg (0.04 mmol, 83% based on Cu[BF_4_]_2_·5H_2_O). IR (KBr, cm^−1^): ν = 2248 (CN). ^1^H NMR (500.3 MHz, acetone-*d*_6_, 298 K, p.p.m.) = 5.12 (*s*, 12H, C_5_H_4_), 4.82 (*s*, 12H, C_5_H_4_). ^13^C{^1^H} NMR: Data not available due to low solubility. HRMS (ESI–TOF): *M* ^+^ C_12_H_8_N_2_CuFe (C_24_H_16_N_4_CuFe_2_): *m*/*z* = 534.9342 (calc. 534.9370); *M* ^+^ C_24_H_16_N_4_CuFe_2_ (C_12_H_8_N_2_CuFe): *m*/*z* = 298.9342 (calc. 298.9333).

## Refinement details   

Crystal data, data collection and structure refinement details are summarized in Table 3 ▸. C-bonded H atoms were placed in calculated positions and constrained to ride on their parent atoms with *U*_iso_(H) = 1.2*U*_eq_(C) and a C—H distance of 0.93 Å for aromatic and *U*_iso_(H) = 1.5*U*_eq_(C) and a C—H distance of 0.96 Å for methyl H atoms. The F atoms of one of the two BF_4_^−^ ions were refined as equally disordered over two sets of sites using DFIX [B—F 1.38 (2) Å] and DANG [F—F 2.25 (4) Å] instructions. Since some anisotropic displacement ellipsoids were rather elongated, DELU/SIMU/ISOR restraints were also applied (McArdle, 1995 ▸; Sheldrick, 2008 ▸).

## Supplementary Material

Crystal structure: contains datablock(s) I. DOI: 10.1107/S2056989015001760/wm5104sup1.cif

Structure factors: contains datablock(s) I. DOI: 10.1107/S2056989015001760/wm5104Isup2.hkl

CCDC reference: 1045804

Additional supporting information:  crystallographic information; 3D view; checkCIF report

## Figures and Tables

**Table 1:** interactions (, ) for (I) The angle is described by calculating the respective bond relative to the centroid of the involved aromatic C_5_ ring.

Involved atoms	distance	
Cu2 C26^A^C27^A^	3.1520(6)	93.23(1)
C26C27 Cu2^B^	3.1520(6)	93.23(1)
C23C23^C^	3.167(6)	92.2(2)

Symmetry codes: (A) *x*1, *y*, *z*; (B) 1+*x*, *y*, *z*; (C) *x*, 1*y*, 1*z*.

**Table 2:** Plane intersection angles () for (I) ^*p*^ defines a plane calculated by the following atom sequence.

CpCp		CpN_3_	
^*p*^(C2C6)^*p*^(C14C18)	11.7(3)	^*p*^(C2C6)^*p*^(N1N3)	11.8(2)
^*p*^(C14C18)^*p*^(C26C30)	23.8(2)	^*p*^(C14C18)^*p*^(N1N3)	18.2(2)
^*p*^(C26C30)^*p*^(C2C6)	13.3(2)	^*p*^(C26C30)^*p*^(N1N3)	8.9(2)
^*p*^(C8C12)^*p*^(C32C36)	12.8(2)	^*p*^(C8C12)^*p*^(N4N6)	8.5(2)
^*p*^(C20C24)^*p*^(C32C36)	23.7(2)	^*p*^(C20C24)^*p*^(N4N6)	19.66(19)
^*p*^(C20C24)^*p*^(C8C12)	11.1(2)	^*p*^(C20C24)^*p*^(N4N6)	5.3(2)

**Table 3:** Experimental details

Crystal data	
Chemical formula	[Cu_2_Fe_3_(C_6_H_4_N)_6_(C_3_H_6_O)](BF_4_)_2_C_3_H_6_O
*M*_r_	1125.02
Crystal system, space group	Triclinic, *P* 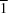
Temperature (K)	110
*a*, *b*, *c* ()	7.9947(6), 13.9384(18), 19.923(2)
, , ()	72.942(10), 82.968(7), 87.936(8)
*V* (^3^)	2106.4(4)
*Z*	2
Radiation type	Cu *K*
(mm^1^)	9.92
Crystal size (mm)	0.4 0.4 0.4

Data collection	
Diffractometer	Oxford Gemini CCD
Absorption correction	Multi-scan (*CrysAlis RED*; Oxford Diffraction, 2006 ▸)
*T*_min_, *T*_max_	0.427, 1.000
No. of measured, independent and observed [*I* > 2(*I*)] reflections	18279, 7318, 6793
*R*_int_	0.042
(sin /)_max_ (^1^)	0.593

Refinement	
*R*[*F* ^2^ > 2(*F* ^2^)], *wR*(*F* ^2^), *S*	0.041, 0.107, 1.05
No. of reflections	7318
No. of parameters	623
No. of restraints	148
H-atom treatment	H-atom parameters constrained
_max_, _min_ (e ^3^)	0.59, 0.49

Computer programs: *CrysAlis CCD* and *CrysAlis RED* (Oxford Diffraction, 2006 ▸), *SHELXS2013* and *SHELXTL* (Sheldrick, 2008 ▸), *SHELXL2013* (Sheldrick, 2015 ▸), *ORTEP-3 for Windows* and *WinGX* (Farrugia, 2012 ▸), *publCIF* (Westrip, 2010 ▸).

## supplementary crystallographic information

### Crystal data

**Table:**

[Cu_2_Fe_3_(C_6_H_4_N)_6_(C_3_H_6_O)](BF_4_)_2_·C_3_H_6_O	*Z* = 2
*M**_r_* = 1125.02	*F*(000) = 1128
Triclinic, *P*1	*D*_x_ = 1.774 Mg m^−^^3^
Hall symbol: -P 1	Cu *K*α radiation, λ = 1.54184 Å
*a* = 7.9947 (6) Å	Cell parameters from 8458 reflections
*b* = 13.9384 (18) Å	θ = 3.3–69.0°
*c* = 19.923 (2) Å	µ = 9.92 mm^−^^1^
α = 72.942 (10)°	*T* = 110 K
β = 82.968 (7)°	Block, orange
γ = 87.936 (8)°	0.4 × 0.4 × 0.4 mm
*V* = 2106.4 (4) Å^3^	

### Data collection

**Table:**

Oxford Gemini CCD diffractometer	7318 independent reflections
Radiation source: fine-focus sealed tube	6793 reflections with *I* > 2σ(*I*)
Graphite monochromator	*R*_int_ = 0.042
ω scans	θ_max_ = 66.0°, θ_min_ = 3.3°
Absorption correction: multi-scan (*CrysAlis RED*; Oxford Diffraction, 2006)	*h* = −9→9
*T*_min_ = 0.427, *T*_max_ = 1.000	*k* = −16→16
18279 measured reflections	*l* = −22→23

### Refinement

**Table:**

Refinement on *F*^2^	Primary atom site location: structure-invariant direct methods
Least-squares matrix: full	Secondary atom site location: difference Fourier map
*R*[*F*^2^ > 2σ(*F*^2^)] = 0.041	Hydrogen site location: inferred from neighbouring sites
*wR*(*F*^2^) = 0.107	H-atom parameters constrained
*S* = 1.05	*w* = 1/[σ^2^(*F*_o_^2^) + (0.0708*P*)^2^ + 0.1006*P*] where *P* = (*F*_o_^2^ + 2*F*_c_^2^)/3
7318 reflections	(Δ/σ)_max_ = 0.001
623 parameters	Δρ_max_ = 0.59 e Å^−^^3^
148 restraints	Δρ_min_ = −0.49 e Å^−^^3^

### Special details

**Table:**

Geometry. All e.s.d.'s (except the e.s.d. in the dihedral angle between two l.s. planes) are estimated using the full covariance matrix. The cell e.s.d.'s are taken into account individually in the estimation of e.s.d.'s in distances, angles and torsion angles; correlations between e.s.d.'s in cell parameters are only used when they are defined by crystal symmetry. An approximate (isotropic) treatment of cell e.s.d.'s is used for estimating e.s.d.'s involving l.s. planes.
Refinement. Refinement of *F*^2^ against ALL reflections. The weighted *R*-factor *wR* and goodness of fit *S* are based on *F*^2^, conventional *R*-factors *R* are based on *F*, with *F* set to zero for negative *F*^2^. The threshold expression of *F*^2^ > σ(*F*^2^) is used only for calculating *R*-factors(gt) *etc*. and is not relevant to the choice of reflections for refinement. *R*-factors based on *F*^2^ are statistically about twice as large as those based on *F*, and *R*- factors based on ALL data will be even larger.

### Fractional atomic coordinates and isotropic or equivalent isotropic displacement parameters (Å^2^)

**Table:**

	*x*	*y*	*z*	*U*_iso_*/*U*_eq_	Occ. (<1)
C1	0.5468 (4)	0.3054 (2)	0.04318 (16)	0.0185 (6)	
C2	0.4808 (4)	0.3038 (2)	−0.01919 (15)	0.0174 (6)	
C3	0.5066 (4)	0.2253 (2)	−0.05327 (16)	0.0200 (6)	
H3	0.5718	0.1678	−0.0393	0.024*	
C4	0.4134 (4)	0.2527 (2)	−0.11210 (15)	0.0220 (6)	
H4	0.4076	0.2160	−0.1439	0.026*	
C5	0.3303 (4)	0.3449 (2)	−0.11466 (16)	0.0221 (6)	
H5	0.2609	0.3782	−0.1484	0.027*	
C6	0.3697 (4)	0.3788 (2)	−0.05765 (16)	0.0202 (6)	
H6	0.3318	0.4372	−0.0472	0.024*	
C7	0.2033 (4)	0.1972 (2)	0.13847 (16)	0.0181 (6)	
C8	0.1377 (4)	0.1837 (2)	0.07864 (15)	0.0179 (6)	
C9	0.1813 (4)	0.1037 (2)	0.04685 (16)	0.0207 (6)	
H9	0.2518	0.0495	0.0631	0.025*	
C10	0.0957 (4)	0.1244 (2)	−0.01372 (17)	0.0219 (6)	
H10	0.0994	0.0848	−0.0442	0.026*	
C11	0.0026 (4)	0.2154 (2)	−0.02100 (16)	0.0227 (6)	
H11	−0.0635	0.2447	−0.0569	0.027*	
C12	0.0277 (4)	0.2537 (2)	0.03579 (16)	0.0202 (6)	
H12	−0.0177	0.3123	0.0438	0.024*	
C13	0.5338 (4)	0.4709 (2)	0.25231 (15)	0.0188 (6)	
C14	0.5060 (4)	0.5502 (2)	0.28328 (15)	0.0182 (6)	
C15	0.5993 (4)	0.5645 (2)	0.33724 (16)	0.0224 (6)	
H15	0.6864	0.5245	0.3573	0.027*	
C16	0.5322 (4)	0.6516 (2)	0.35356 (18)	0.0282 (8)	
H16	0.5690	0.6791	0.3865	0.034*	
C17	0.3998 (5)	0.6902 (2)	0.31161 (18)	0.0288 (8)	
H17	0.3355	0.7468	0.3126	0.035*	
C18	0.3822 (4)	0.6281 (2)	0.26801 (16)	0.0225 (7)	
H18	0.3048	0.6364	0.2355	0.027*	
C19	0.2308 (4)	0.3458 (2)	0.36694 (15)	0.0172 (6)	
C20	0.2118 (4)	0.4172 (2)	0.40581 (15)	0.0164 (6)	
C21	0.3173 (4)	0.4224 (2)	0.45839 (15)	0.0179 (6)	
H21	0.4010	0.3771	0.4759	0.022*	
C22	0.2683 (4)	0.5103 (2)	0.47808 (15)	0.0197 (6)	
H22	0.3150	0.5324	0.5114	0.024*	
C23	0.1375 (4)	0.5588 (2)	0.43916 (17)	0.0218 (6)	
H23	0.0844	0.6179	0.4426	0.026*	
C24	0.1004 (4)	0.5022 (2)	0.39356 (16)	0.0202 (6)	
H24	0.0198	0.5174	0.3621	0.024*	
C25	0.8920 (4)	0.1538 (2)	0.27510 (15)	0.0161 (6)	
C26	0.9887 (4)	0.0779 (2)	0.31755 (15)	0.0160 (6)	
C27	1.0231 (4)	0.0708 (2)	0.38812 (16)	0.0180 (6)	
H27	0.9901	0.1161	0.4134	0.022*	
C28	1.1172 (4)	−0.0189 (2)	0.41175 (16)	0.0170 (6)	
H28	1.1573	−0.0423	0.4556	0.020*	
C29	1.1401 (4)	−0.0672 (2)	0.35708 (16)	0.0188 (6)	
H29	1.1974	−0.1271	0.3595	0.023*	
C30	1.0613 (4)	−0.0089 (2)	0.29883 (16)	0.0181 (6)	
H30	1.0568	−0.0234	0.2564	0.022*	
C31	0.5433 (4)	0.0322 (2)	0.34912 (16)	0.0173 (6)	
C32	0.6342 (4)	−0.0514 (2)	0.38813 (16)	0.0171 (6)	
C33	0.6779 (4)	−0.0651 (2)	0.45801 (16)	0.0205 (6)	
H33	0.6474	−0.0236	0.4865	0.025*	
C34	0.7752 (4)	−0.1528 (2)	0.47542 (16)	0.0239 (7)	
H34	0.8195	−0.1799	0.5180	0.029*	
C35	0.7953 (4)	−0.1936 (2)	0.41705 (18)	0.0233 (7)	
H35	0.8553	−0.2514	0.4152	0.028*	
C36	0.7084 (4)	−0.1314 (2)	0.36238 (16)	0.0199 (6)	
H36	0.7009	−0.1405	0.3184	0.024*	
C37	1.1665 (5)	0.5108 (3)	0.0935 (2)	0.0435 (10)	
H37A	1.1566	0.5118	0.0457	0.065*	
H37B	1.2669	0.4751	0.1083	0.065*	
H37C	1.1726	0.5783	0.0959	0.065*	
C38	1.0157 (4)	0.4592 (2)	0.14111 (18)	0.0223 (6)	
C39	1.0100 (5)	0.4503 (3)	0.2177 (2)	0.0332 (8)	
H39A	0.9086	0.4161	0.2428	0.050*	
H39B	1.0117	0.5160	0.2238	0.050*	
H39C	1.1061	0.4129	0.2359	0.050*	
C40	0.4543 (4)	−0.0511 (2)	0.19261 (17)	0.0247 (7)	
H40A	0.3626	−0.0733	0.1740	0.037*	
H40B	0.4127	−0.0055	0.2187	0.037*	
H40C	0.5052	−0.1080	0.2234	0.037*	
C41	0.5833 (4)	0.0012 (2)	0.13277 (17)	0.0223 (7)	
C42	0.7365 (5)	0.0406 (3)	0.1520 (2)	0.0313 (8)	
H42A	0.8095	0.0720	0.1098	0.047*	
H42B	0.7950	−0.0137	0.1817	0.047*	
H42C	0.7034	0.0890	0.1769	0.047*	
N1	0.6008 (3)	0.30829 (19)	0.09341 (14)	0.0196 (5)	
N2	0.5629 (3)	0.40839 (19)	0.22584 (13)	0.0216 (5)	
N3	0.8167 (3)	0.21500 (17)	0.23846 (13)	0.0179 (5)	
N4	0.2546 (3)	0.20846 (19)	0.18660 (13)	0.0202 (5)	
N5	0.2515 (3)	0.29064 (18)	0.33446 (13)	0.0196 (5)	
N6	0.4698 (3)	0.09815 (19)	0.31814 (14)	0.0204 (5)	
Fe1	0.25591 (6)	0.24050 (3)	−0.02036 (2)	0.01424 (12)	
Fe2	0.34679 (5)	0.54574 (3)	0.37176 (2)	0.01361 (12)	
Fe3	0.88811 (5)	−0.05035 (3)	0.38612 (2)	0.01260 (12)	
Cu1	0.68186 (5)	0.31536 (3)	0.17936 (2)	0.01732 (12)	
Cu2	0.32370 (5)	0.20644 (3)	0.27549 (2)	0.01731 (12)	
O1	0.9045 (3)	0.42672 (16)	0.11721 (11)	0.0229 (5)	
O2	0.5640 (3)	0.0100 (2)	0.07178 (13)	0.0372 (6)	
F1	−0.030 (3)	0.3112 (14)	0.8055 (12)	0.053 (5)	0.50
F2	0.1898 (14)	0.2122 (16)	0.7813 (11)	0.037 (3)	0.50
F3	−0.031 (2)	0.2441 (11)	0.7154 (7)	0.072 (3)	0.50
F4	−0.083 (2)	0.1484 (11)	0.8103 (7)	0.077 (4)	0.50
F1'	−0.015 (3)	0.3066 (11)	0.8136 (11)	0.033 (2)	0.50
F2'	0.1857 (15)	0.1996 (15)	0.7868 (11)	0.040 (4)	0.50
F3'	0.000 (2)	0.2664 (12)	0.7105 (5)	0.094 (5)	0.50
F4'	−0.062 (2)	0.1452 (9)	0.8301 (6)	0.050 (2)	0.50
F5	0.8995 (3)	0.25555 (17)	0.46188 (15)	0.0480 (6)	
F6	0.6375 (3)	0.26511 (19)	0.51557 (14)	0.0479 (6)	
F7	0.6937 (5)	0.3401 (2)	0.39964 (16)	0.0729 (9)	
F8	0.6880 (3)	0.17002 (17)	0.44066 (14)	0.0445 (6)	
B1	0.0191 (5)	0.2297 (3)	0.7814 (2)	0.0320 (9)	
B2	0.7293 (5)	0.2580 (3)	0.4537 (2)	0.0270 (8)	

### Atomic displacement parameters (Å^2^)

**Table:**

	*U*^11^	*U*^22^	*U*^33^	*U*^12^	*U*^13^	*U*^23^
C1	0.0139 (14)	0.0180 (14)	0.0234 (16)	−0.0030 (11)	0.0033 (12)	−0.0076 (11)
C2	0.0149 (14)	0.0196 (14)	0.0176 (14)	−0.0046 (11)	0.0017 (11)	−0.0063 (11)
C3	0.0185 (15)	0.0218 (15)	0.0200 (15)	−0.0030 (12)	0.0044 (12)	−0.0089 (12)
C4	0.0248 (16)	0.0269 (16)	0.0152 (14)	−0.0084 (13)	0.0054 (12)	−0.0096 (12)
C5	0.0252 (16)	0.0232 (15)	0.0143 (14)	−0.0065 (12)	−0.0011 (12)	0.0007 (11)
C6	0.0223 (15)	0.0171 (14)	0.0196 (15)	−0.0049 (12)	0.0016 (12)	−0.0039 (11)
C7	0.0163 (14)	0.0168 (14)	0.0189 (15)	−0.0050 (11)	0.0042 (12)	−0.0035 (11)
C8	0.0162 (14)	0.0215 (14)	0.0144 (14)	−0.0068 (11)	0.0038 (11)	−0.0041 (11)
C9	0.0229 (16)	0.0164 (14)	0.0225 (15)	−0.0083 (12)	0.0009 (12)	−0.0056 (11)
C10	0.0217 (16)	0.0228 (15)	0.0246 (16)	−0.0100 (12)	−0.0005 (12)	−0.0119 (12)
C11	0.0165 (15)	0.0300 (16)	0.0212 (15)	−0.0064 (12)	−0.0018 (12)	−0.0063 (12)
C12	0.0141 (14)	0.0239 (15)	0.0224 (15)	−0.0031 (11)	0.0035 (11)	−0.0083 (12)
C13	0.0171 (14)	0.0176 (14)	0.0182 (14)	−0.0028 (11)	0.0065 (11)	−0.0026 (12)
C14	0.0207 (15)	0.0153 (13)	0.0163 (14)	−0.0039 (11)	0.0073 (11)	−0.0044 (11)
C15	0.0158 (15)	0.0268 (16)	0.0240 (15)	−0.0105 (12)	0.0051 (12)	−0.0083 (12)
C16	0.0331 (19)	0.0252 (16)	0.0265 (16)	−0.0184 (14)	0.0137 (14)	−0.0128 (13)
C17	0.041 (2)	0.0073 (13)	0.0327 (17)	−0.0044 (13)	0.0137 (15)	−0.0037 (12)
C18	0.0320 (17)	0.0141 (14)	0.0158 (14)	0.0019 (12)	0.0071 (12)	0.0005 (11)
C19	0.0202 (15)	0.0118 (13)	0.0171 (14)	−0.0055 (11)	0.0008 (11)	−0.0009 (11)
C20	0.0179 (14)	0.0119 (13)	0.0180 (14)	−0.0063 (11)	0.0042 (11)	−0.0039 (10)
C21	0.0236 (15)	0.0149 (13)	0.0131 (13)	−0.0043 (11)	0.0011 (11)	−0.0014 (10)
C22	0.0269 (16)	0.0185 (14)	0.0134 (13)	−0.0099 (12)	0.0065 (12)	−0.0065 (11)
C23	0.0197 (15)	0.0167 (14)	0.0291 (16)	−0.0010 (11)	0.0090 (12)	−0.0113 (12)
C24	0.0147 (14)	0.0201 (14)	0.0254 (15)	−0.0026 (11)	0.0018 (12)	−0.0072 (12)
C25	0.0156 (14)	0.0105 (13)	0.0226 (14)	−0.0066 (11)	0.0050 (12)	−0.0074 (11)
C26	0.0145 (14)	0.0118 (13)	0.0204 (14)	−0.0064 (11)	0.0033 (11)	−0.0040 (11)
C27	0.0168 (14)	0.0149 (13)	0.0238 (15)	−0.0047 (11)	0.0013 (11)	−0.0088 (11)
C28	0.0110 (13)	0.0158 (13)	0.0238 (15)	−0.0037 (10)	−0.0027 (11)	−0.0044 (11)
C29	0.0145 (14)	0.0123 (13)	0.0283 (16)	0.0006 (11)	0.0029 (12)	−0.0063 (11)
C30	0.0160 (14)	0.0157 (14)	0.0217 (14)	−0.0053 (11)	0.0077 (11)	−0.0074 (11)
C31	0.0118 (13)	0.0190 (15)	0.0225 (15)	−0.0052 (12)	0.0020 (11)	−0.0091 (12)
C32	0.0142 (14)	0.0139 (13)	0.0223 (15)	−0.0049 (11)	−0.0013 (11)	−0.0037 (11)
C33	0.0145 (14)	0.0245 (15)	0.0207 (15)	−0.0086 (12)	0.0040 (11)	−0.0052 (12)
C34	0.0195 (15)	0.0244 (16)	0.0204 (15)	−0.0125 (12)	−0.0016 (12)	0.0061 (12)
C35	0.0237 (16)	0.0079 (13)	0.0345 (17)	−0.0067 (11)	−0.0041 (13)	0.0007 (12)
C36	0.0209 (15)	0.0146 (14)	0.0256 (15)	−0.0085 (11)	−0.0032 (12)	−0.0069 (11)
C37	0.033 (2)	0.035 (2)	0.061 (3)	−0.0126 (16)	0.0047 (19)	−0.0136 (18)
C38	0.0209 (16)	0.0117 (13)	0.0349 (17)	0.0032 (11)	−0.0052 (13)	−0.0072 (12)
C39	0.042 (2)	0.0217 (16)	0.038 (2)	−0.0033 (14)	−0.0168 (16)	−0.0066 (14)
C40	0.0247 (17)	0.0257 (16)	0.0241 (16)	−0.0031 (13)	0.0010 (13)	−0.0089 (12)
C41	0.0249 (16)	0.0146 (14)	0.0269 (17)	0.0030 (12)	−0.0007 (13)	−0.0068 (12)
C42	0.0324 (19)	0.0250 (17)	0.0369 (19)	−0.0083 (14)	−0.0026 (15)	−0.0092 (14)
N1	0.0155 (12)	0.0221 (13)	0.0229 (13)	−0.0025 (10)	−0.0018 (10)	−0.0090 (10)
N2	0.0237 (14)	0.0190 (12)	0.0215 (13)	0.0030 (10)	0.0030 (10)	−0.0074 (10)
N3	0.0210 (13)	0.0127 (12)	0.0197 (12)	−0.0035 (10)	0.0003 (10)	−0.0051 (10)
N4	0.0203 (13)	0.0214 (13)	0.0182 (13)	−0.0024 (10)	0.0016 (10)	−0.0058 (10)
N5	0.0236 (13)	0.0154 (12)	0.0194 (12)	−0.0031 (10)	−0.0006 (10)	−0.0050 (10)
N6	0.0160 (12)	0.0210 (13)	0.0256 (13)	0.0004 (10)	−0.0021 (10)	−0.0093 (11)
Fe1	0.0156 (2)	0.0142 (2)	0.0135 (2)	−0.00296 (17)	0.00021 (17)	−0.00528 (17)
Fe2	0.0160 (2)	0.0091 (2)	0.0157 (2)	−0.00264 (16)	0.00292 (17)	−0.00522 (16)
Fe3	0.0128 (2)	0.0089 (2)	0.0161 (2)	−0.00297 (16)	−0.00034 (17)	−0.00383 (16)
Cu1	0.0201 (2)	0.0150 (2)	0.0186 (2)	0.00069 (16)	−0.00220 (17)	−0.00767 (16)
Cu2	0.0195 (2)	0.0161 (2)	0.0179 (2)	−0.00040 (16)	−0.00213 (17)	−0.00739 (16)
O1	0.0236 (11)	0.0205 (10)	0.0259 (11)	−0.0038 (9)	−0.0005 (9)	−0.0093 (8)
O2	0.0411 (15)	0.0443 (15)	0.0246 (13)	−0.0013 (12)	−0.0053 (11)	−0.0067 (11)
F1	0.085 (9)	0.046 (6)	0.048 (8)	0.032 (4)	−0.046 (5)	−0.035 (6)
F2	0.027 (4)	0.044 (6)	0.034 (5)	−0.008 (3)	0.000 (4)	−0.006 (4)
F3	0.090 (7)	0.079 (5)	0.086 (6)	0.045 (4)	−0.065 (5)	−0.069 (4)
F4	0.037 (5)	0.090 (6)	0.136 (10)	−0.027 (5)	0.011 (7)	−0.088 (6)
F1'	0.056 (5)	0.022 (4)	0.029 (4)	0.014 (4)	−0.018 (6)	−0.015 (3)
F2'	0.037 (5)	0.044 (6)	0.055 (9)	0.015 (4)	−0.025 (5)	−0.034 (6)
F3'	0.116 (10)	0.155 (12)	0.035 (4)	0.107 (9)	−0.053 (4)	−0.059 (5)
F4'	0.045 (5)	0.043 (3)	0.074 (5)	−0.018 (3)	−0.010 (4)	−0.032 (3)
F5	0.0255 (11)	0.0393 (12)	0.0848 (18)	−0.0003 (9)	−0.0047 (11)	−0.0275 (12)
F6	0.0439 (13)	0.0476 (13)	0.0608 (15)	−0.0020 (10)	0.0085 (11)	−0.0348 (11)
F7	0.108 (3)	0.0435 (15)	0.0600 (17)	0.0378 (16)	−0.0194 (16)	−0.0047 (12)
F8	0.0368 (12)	0.0356 (11)	0.0757 (16)	0.0038 (9)	−0.0083 (11)	−0.0387 (11)
B1	0.038 (2)	0.034 (2)	0.036 (2)	0.0115 (17)	−0.0196 (18)	−0.0245 (17)
B2	0.0236 (19)	0.0181 (17)	0.041 (2)	0.0068 (14)	−0.0013 (16)	−0.0125 (15)

### Geometric parameters (Å, º)

**Table:**

C1—N1	1.149 (4)	C26—C30	1.449 (4)
C1—C2	1.414 (4)	C26—Fe3	2.027 (3)
C2—C3	1.444 (4)	C27—C28	1.423 (4)
C2—C6	1.449 (4)	C27—Fe3	2.049 (3)
C2—Fe1	2.037 (3)	C27—H27	0.9300
C3—C4	1.416 (5)	C28—C29	1.430 (4)
C3—Fe1	2.054 (3)	C28—Fe3	2.056 (3)
C3—H3	0.9300	C28—H28	0.9300
C4—C5	1.416 (5)	C29—C30	1.412 (5)
C4—Fe1	2.055 (3)	C29—Fe3	2.051 (3)
C4—H4	0.9300	C29—H29	0.9300
C5—C6	1.423 (5)	C30—Fe3	2.038 (3)
C5—Fe1	2.046 (3)	C30—H30	0.9300
C5—H5	0.9300	C31—N6	1.135 (4)
C6—Fe1	2.053 (3)	C31—C32	1.426 (4)
C6—H6	0.9300	C32—C33	1.434 (4)
C7—N4	1.141 (4)	C32—C36	1.441 (4)
C7—C8	1.422 (4)	C32—Fe3	2.026 (3)
C8—C12	1.445 (5)	C33—C34	1.403 (5)
C8—C9	1.448 (4)	C33—Fe3	2.045 (3)
C8—Fe1	2.023 (3)	C33—H33	0.9300
C9—C10	1.411 (5)	C34—C35	1.428 (5)
C9—Fe1	2.043 (3)	C34—Fe3	2.056 (3)
C9—H9	0.9300	C34—H34	0.9300
C10—C11	1.425 (5)	C35—C36	1.417 (5)
C10—Fe1	2.064 (3)	C35—Fe3	2.047 (3)
C10—H10	0.9300	C35—H35	0.9300
C11—C12	1.423 (5)	C36—Fe3	2.040 (3)
C11—Fe1	2.070 (3)	C36—H36	0.9300
C11—H11	0.9300	C37—C38	1.501 (5)
C12—Fe1	2.052 (3)	C37—H37A	0.9600
C12—H12	0.9300	C37—H37B	0.9600
C13—N2	1.148 (4)	C37—H37C	0.9600
C13—C14	1.414 (4)	C38—O1	1.217 (4)
C14—C18	1.434 (4)	C38—C39	1.489 (5)
C14—C15	1.444 (5)	C39—H39A	0.9600
C14—Fe2	2.031 (3)	C39—H39B	0.9600
C15—C16	1.417 (5)	C39—H39C	0.9600
C15—Fe2	2.052 (3)	C40—C41	1.503 (4)
C15—H15	0.9300	C40—H40A	0.9600
C16—C17	1.422 (6)	C40—H40B	0.9600
C16—Fe2	2.054 (3)	C40—H40C	0.9600
C16—H16	0.9300	C41—O2	1.214 (4)
C17—C18	1.415 (5)	C41—C42	1.496 (5)
C17—Fe2	2.048 (3)	C42—H42A	0.9600
C17—H17	0.9300	C42—H42B	0.9600
C18—Fe2	2.039 (3)	C42—H42C	0.9600
C18—H18	0.9300	N1—Cu1	1.933 (3)
C19—N5	1.137 (4)	N2—Cu1	1.960 (3)
C19—C20	1.423 (4)	N3—Cu1	1.934 (3)
C20—C24	1.437 (4)	N4—Cu2	1.911 (3)
C20—C21	1.442 (4)	N5—Cu2	1.920 (3)
C20—Fe2	2.021 (3)	N6—Cu2	1.931 (3)
C21—C22	1.421 (4)	Cu1—O1	2.375 (2)
C21—Fe2	2.044 (3)	F1—B1	1.385 (13)
C21—H21	0.9300	F2—B1	1.378 (12)
C22—C23	1.411 (5)	F3—B1	1.378 (11)
C22—Fe2	2.051 (3)	F4—B1	1.362 (12)
C22—H22	0.9300	F1'—B1	1.404 (11)
C23—C24	1.429 (4)	F2'—B1	1.389 (12)
C23—Fe2	2.051 (3)	F3'—B1	1.378 (9)
C23—H23	0.9300	F4'—B1	1.407 (10)
C24—Fe2	2.039 (3)	F5—B2	1.388 (5)
C24—H24	0.9300	F6—B2	1.382 (5)
C25—N3	1.152 (4)	F7—B2	1.371 (5)
C25—C26	1.417 (4)	F8—B2	1.385 (4)
C26—C27	1.440 (4)		
			
N1—C1—C2	178.9 (3)	C41—C40—H40A	109.5
C1—C2—C3	125.8 (3)	C41—C40—H40B	109.5
C1—C2—C6	125.4 (3)	H40A—C40—H40B	109.5
C3—C2—C6	108.7 (3)	C41—C40—H40C	109.5
C1—C2—Fe1	124.0 (2)	H40A—C40—H40C	109.5
C3—C2—Fe1	69.98 (17)	H40B—C40—H40C	109.5
C6—C2—Fe1	69.87 (17)	O2—C41—C42	121.8 (3)
C4—C3—C2	106.7 (3)	O2—C41—C40	121.2 (3)
C4—C3—Fe1	69.87 (18)	C42—C41—C40	117.0 (3)
C2—C3—Fe1	68.67 (17)	C41—C42—H42A	109.5
C4—C3—H3	126.6	C41—C42—H42B	109.5
C2—C3—H3	126.6	H42A—C42—H42B	109.5
Fe1—C3—H3	126.4	C41—C42—H42C	109.5
C5—C4—C3	109.0 (3)	H42A—C42—H42C	109.5
C5—C4—Fe1	69.46 (17)	H42B—C42—H42C	109.5
C3—C4—Fe1	69.81 (16)	C1—N1—Cu1	177.4 (2)
C5—C4—H4	125.5	C13—N2—Cu1	162.7 (3)
C3—C4—H4	125.5	C25—N3—Cu1	177.6 (2)
Fe1—C4—H4	126.8	C7—N4—Cu2	170.5 (2)
C4—C5—C6	109.4 (3)	C19—N5—Cu2	170.5 (3)
C4—C5—Fe1	70.15 (16)	C31—N6—Cu2	172.9 (2)
C6—C5—Fe1	69.96 (16)	C8—Fe1—C2	111.30 (12)
C4—C5—H5	125.3	C8—Fe1—C9	41.73 (12)
C6—C5—H5	125.3	C2—Fe1—C9	122.57 (13)
Fe1—C5—H5	126.2	C8—Fe1—C5	158.37 (13)
C5—C6—C2	106.1 (3)	C2—Fe1—C5	68.38 (12)
C5—C6—Fe1	69.42 (16)	C9—Fe1—C5	157.48 (13)
C2—C6—Fe1	68.65 (16)	C8—Fe1—C12	41.54 (13)
C5—C6—H6	127.0	C2—Fe1—C12	128.56 (12)
C2—C6—H6	127.0	C9—Fe1—C12	70.17 (13)
Fe1—C6—H6	126.5	C5—Fe1—C12	120.54 (13)
N4—C7—C8	179.4 (3)	C8—Fe1—C6	124.44 (12)
C7—C8—C12	124.8 (3)	C2—Fe1—C6	41.49 (13)
C7—C8—C9	126.1 (3)	C9—Fe1—C6	159.97 (13)
C12—C8—C9	108.9 (3)	C5—Fe1—C6	40.62 (13)
C7—C8—Fe1	121.6 (2)	C12—Fe1—C6	109.01 (13)
C12—C8—Fe1	70.32 (16)	C8—Fe1—C3	126.63 (13)
C9—C8—Fe1	69.87 (16)	C2—Fe1—C3	41.35 (12)
C10—C9—C8	106.4 (3)	C9—Fe1—C3	105.80 (13)
C10—C9—Fe1	70.70 (17)	C5—Fe1—C3	68.44 (13)
C8—C9—Fe1	68.40 (16)	C12—Fe1—C3	165.74 (13)
C10—C9—H9	126.8	C6—Fe1—C3	69.84 (12)
C8—C9—H9	126.8	C8—Fe1—C4	161.08 (13)
Fe1—C9—H9	125.7	C2—Fe1—C4	68.25 (12)
C9—C10—C11	109.5 (3)	C9—Fe1—C4	121.35 (13)
C9—C10—Fe1	69.10 (16)	C5—Fe1—C4	40.39 (13)
C11—C10—Fe1	70.05 (17)	C12—Fe1—C4	153.52 (13)
C9—C10—H10	125.2	C6—Fe1—C4	68.67 (12)
C11—C10—H10	125.2	C3—Fe1—C4	40.32 (13)
Fe1—C10—H10	127.2	C8—Fe1—C10	68.17 (12)
C12—C11—C10	108.7 (3)	C2—Fe1—C10	155.50 (13)
C12—C11—Fe1	69.15 (17)	C9—Fe1—C10	40.20 (13)
C10—C11—Fe1	69.60 (18)	C5—Fe1—C10	121.64 (13)
C12—C11—H11	125.6	C12—Fe1—C10	68.45 (12)
C10—C11—H11	125.6	C6—Fe1—C10	159.38 (13)
Fe1—C11—H11	127.2	C3—Fe1—C10	117.83 (13)
C11—C12—C8	106.5 (3)	C4—Fe1—C10	104.04 (12)
C11—C12—Fe1	70.46 (17)	C8—Fe1—C11	68.30 (12)
C8—C12—Fe1	68.14 (16)	C2—Fe1—C11	163.98 (13)
C11—C12—H12	126.8	C9—Fe1—C11	68.58 (13)
C8—C12—H12	126.8	C5—Fe1—C11	105.79 (13)
Fe1—C12—H12	126.2	C12—Fe1—C11	40.39 (13)
N2—C13—C14	177.1 (3)	C6—Fe1—C11	124.52 (13)
C13—C14—C18	126.9 (3)	C3—Fe1—C11	152.10 (13)
C13—C14—C15	124.6 (3)	C4—Fe1—C11	117.67 (13)
C18—C14—C15	108.6 (3)	C10—Fe1—C11	40.35 (13)
C13—C14—Fe2	125.5 (2)	C20—Fe2—C14	111.25 (12)
C18—C14—Fe2	69.70 (16)	C20—Fe2—C18	122.55 (13)
C15—C14—Fe2	70.07 (16)	C14—Fe2—C18	41.24 (13)
C16—C15—C14	106.5 (3)	C20—Fe2—C24	41.45 (12)
C16—C15—Fe2	69.89 (18)	C14—Fe2—C24	127.62 (13)
C14—C15—Fe2	68.51 (16)	C18—Fe2—C24	106.96 (14)
C16—C15—H15	126.7	C20—Fe2—C21	41.55 (12)
C14—C15—H15	126.7	C14—Fe2—C21	123.44 (12)
Fe2—C15—H15	126.4	C18—Fe2—C21	159.02 (12)
C15—C16—C17	109.1 (3)	C24—Fe2—C21	69.95 (12)
C15—C16—Fe2	69.74 (17)	C20—Fe2—C17	155.36 (15)
C17—C16—Fe2	69.48 (18)	C14—Fe2—C17	68.43 (12)
C15—C16—H16	125.4	C18—Fe2—C17	40.52 (14)
C17—C16—H16	125.4	C24—Fe2—C17	117.82 (14)
Fe2—C16—H16	126.9	C21—Fe2—C17	159.70 (14)
C18—C17—C16	108.6 (3)	C20—Fe2—C23	68.72 (12)
C18—C17—Fe2	69.43 (16)	C14—Fe2—C23	162.87 (14)
C16—C17—Fe2	69.96 (18)	C18—Fe2—C23	123.29 (13)
C18—C17—H17	125.7	C24—Fe2—C23	40.88 (13)
C16—C17—H17	125.7	C21—Fe2—C23	68.70 (12)
Fe2—C17—H17	126.5	C17—Fe2—C23	104.13 (13)
C17—C18—C14	107.2 (3)	C20—Fe2—C22	68.55 (12)
C17—C18—Fe2	70.06 (17)	C14—Fe2—C22	156.78 (14)
C14—C18—Fe2	69.06 (16)	C18—Fe2—C22	159.14 (13)
C17—C18—H18	126.4	C24—Fe2—C22	68.68 (13)
C14—C18—H18	126.4	C21—Fe2—C22	40.59 (12)
Fe2—C18—H18	126.1	C17—Fe2—C22	122.00 (13)
N5—C19—C20	177.4 (3)	C23—Fe2—C22	40.24 (14)
C19—C20—C24	126.1 (3)	C20—Fe2—C15	128.54 (13)
C19—C20—C21	124.7 (3)	C14—Fe2—C15	41.43 (13)
C24—C20—C21	108.8 (3)	C18—Fe2—C15	69.64 (14)
C19—C20—Fe2	120.2 (2)	C24—Fe2—C15	166.46 (13)
C24—C20—Fe2	69.97 (16)	C21—Fe2—C15	108.26 (13)
C21—C20—Fe2	70.09 (16)	C17—Fe2—C15	68.68 (14)
C22—C21—C20	106.5 (3)	C23—Fe2—C15	152.02 (13)
C22—C21—Fe2	69.97 (16)	C22—Fe2—C15	119.25 (13)
C20—C21—Fe2	68.35 (15)	C20—Fe2—C16	163.92 (15)
C22—C21—H21	126.8	C14—Fe2—C16	68.29 (12)
C20—C21—H21	126.8	C18—Fe2—C16	68.48 (14)
Fe2—C21—H21	126.5	C24—Fe2—C16	151.81 (14)
C23—C22—C21	109.4 (3)	C21—Fe2—C16	124.25 (14)
C23—C22—Fe2	69.87 (17)	C17—Fe2—C16	40.56 (16)
C21—C22—Fe2	69.44 (16)	C23—Fe2—C16	116.77 (13)
C23—C22—H22	125.3	C22—Fe2—C16	105.22 (12)
C21—C22—H22	125.3	C15—Fe2—C16	40.37 (14)
Fe2—C22—H22	127.0	C32—Fe3—C26	111.01 (12)
C22—C23—C24	108.7 (3)	C32—Fe3—C30	126.88 (13)
C22—C23—Fe2	69.89 (17)	C26—Fe3—C30	41.77 (12)
C24—C23—Fe2	69.12 (17)	C32—Fe3—C36	41.50 (12)
C22—C23—H23	125.6	C26—Fe3—C36	123.05 (12)
C24—C23—H23	125.6	C30—Fe3—C36	106.54 (12)
Fe2—C23—H23	126.9	C32—Fe3—C33	41.23 (13)
C23—C24—C20	106.7 (3)	C26—Fe3—C33	127.68 (12)
C23—C24—Fe2	69.99 (17)	C30—Fe3—C33	165.43 (13)
C20—C24—Fe2	68.58 (16)	C36—Fe3—C33	69.70 (12)
C23—C24—H24	126.7	C32—Fe3—C35	68.46 (12)
C20—C24—H24	126.7	C26—Fe3—C35	156.48 (13)
Fe2—C24—H24	126.3	C30—Fe3—C35	118.37 (13)
N3—C25—C26	177.5 (3)	C36—Fe3—C35	40.57 (13)
C25—C26—C27	125.8 (3)	C33—Fe3—C35	68.51 (13)
C25—C26—C30	125.7 (3)	C32—Fe3—C27	124.00 (12)
C27—C26—C30	108.4 (3)	C26—Fe3—C27	41.37 (12)
C25—C26—Fe3	123.5 (2)	C30—Fe3—C27	69.99 (12)
C27—C26—Fe3	70.12 (15)	C36—Fe3—C27	159.91 (13)
C30—C26—Fe3	69.51 (15)	C33—Fe3—C27	108.45 (12)
C28—C27—C26	106.8 (3)	C35—Fe3—C27	158.82 (13)
C28—C27—Fe3	69.97 (16)	C32—Fe3—C29	161.44 (13)
C26—C27—Fe3	68.51 (16)	C26—Fe3—C29	68.70 (12)
C28—C27—H27	126.6	C30—Fe3—C29	40.39 (13)
C26—C27—H27	126.6	C36—Fe3—C29	121.97 (12)
Fe3—C27—H27	126.5	C33—Fe3—C29	153.64 (13)
C27—C28—C29	108.8 (3)	C35—Fe3—C29	104.07 (12)
C27—C28—Fe3	69.46 (16)	C27—Fe3—C29	68.91 (12)
C29—C28—Fe3	69.45 (17)	C32—Fe3—C28	157.65 (12)
C27—C28—H28	125.6	C26—Fe3—C28	68.54 (12)
C29—C28—H28	125.6	C30—Fe3—C28	68.72 (12)
Fe3—C28—H28	127.1	C36—Fe3—C28	158.11 (12)
C30—C29—C28	108.8 (3)	C33—Fe3—C28	120.10 (12)
C30—C29—Fe3	69.30 (16)	C35—Fe3—C28	121.38 (12)
C28—C29—Fe3	69.80 (16)	C27—Fe3—C28	40.57 (12)
C30—C29—H29	125.6	C29—Fe3—C28	40.75 (12)
C28—C29—H29	125.6	C32—Fe3—C34	68.04 (12)
Fe3—C29—H29	126.9	C26—Fe3—C34	162.55 (13)
C29—C30—C26	107.1 (3)	C30—Fe3—C34	153.04 (13)
C29—C30—Fe3	70.31 (16)	C36—Fe3—C34	68.66 (13)
C26—C30—Fe3	68.72 (15)	C33—Fe3—C34	40.01 (13)
C29—C30—H30	126.4	C35—Fe3—C34	40.72 (14)
C26—C30—H30	126.4	C27—Fe3—C34	123.56 (13)
Fe3—C30—H30	126.1	C29—Fe3—C34	118.09 (13)
N6—C31—C32	179.4 (3)	C28—Fe3—C34	105.38 (12)
C31—C32—C33	125.2 (3)	N1—Cu1—N3	126.02 (11)
C31—C32—C36	126.1 (3)	N1—Cu1—N2	116.24 (11)
C33—C32—C36	108.6 (3)	N3—Cu1—N2	114.92 (11)
C31—C32—Fe3	122.43 (19)	N1—Cu1—O1	93.09 (9)
C33—C32—Fe3	70.08 (17)	N3—Cu1—O1	97.54 (9)
C36—C32—Fe3	69.77 (17)	N2—Cu1—O1	96.17 (9)
C34—C33—C32	107.3 (3)	N4—Cu2—N5	128.97 (11)
C34—C33—Fe3	70.43 (17)	N4—Cu2—N6	117.11 (11)
C32—C33—Fe3	68.69 (16)	N5—Cu2—N6	113.63 (11)
C34—C33—H33	126.4	C38—O1—Cu1	128.4 (2)
C32—C33—H33	126.4	F4—B1—F3	92.2 (9)
Fe3—C33—H33	126.1	F4—B1—F2	116.4 (12)
C33—C34—C35	108.9 (3)	F3—B1—F2	111.8 (12)
C33—C34—Fe3	69.56 (16)	F4—B1—F3'	108.4 (10)
C35—C34—Fe3	69.30 (16)	F3—B1—F3'	16.2 (13)
C33—C34—H34	125.5	F2—B1—F3'	103.2 (12)
C35—C34—H34	125.5	F4—B1—F1	113.4 (12)
Fe3—C34—H34	127.2	F3—B1—F1	109.4 (13)
C36—C35—C34	108.6 (3)	F2—B1—F1	112.0 (14)
C36—C35—Fe3	69.45 (16)	F3'—B1—F1	101.9 (13)
C34—C35—Fe3	69.97 (17)	F4—B1—F2'	109.0 (12)
C36—C35—H35	125.7	F3—B1—F2'	114.6 (13)
C34—C35—H35	125.7	F2—B1—F2'	7.4 (16)
Fe3—C35—H35	126.5	F3'—B1—F2'	107.6 (10)
C35—C36—C32	106.6 (3)	F1—B1—F2'	115.9 (16)
C35—C36—Fe3	69.99 (17)	F4—B1—F1'	113.5 (11)
C32—C36—Fe3	68.74 (16)	F3—B1—F1'	117.8 (13)
C35—C36—H36	126.7	F2—B1—F1'	105.5 (15)
C32—C36—H36	126.7	F3'—B1—F1'	109.3 (11)
Fe3—C36—H36	126.1	F1—B1—F1'	9 (2)
C38—C37—H37A	109.5	F2'—B1—F1'	108.8 (12)
C38—C37—H37B	109.5	F4—B1—F4'	18.1 (9)
H37A—C37—H37B	109.5	F3—B1—F4'	110.3 (8)
C38—C37—H37C	109.5	F2—B1—F4'	106.7 (11)
H37A—C37—H37C	109.5	F3'—B1—F4'	126.5 (10)
H37B—C37—H37C	109.5	F1—B1—F4'	106.5 (13)
O1—C38—C39	122.5 (3)	F2'—B1—F4'	99.3 (11)
O1—C38—C37	120.6 (3)	F1'—B1—F4'	104.1 (9)
C39—C38—C37	116.9 (3)	F7—B2—F6	108.5 (3)
C38—C39—H39A	109.5	F7—B2—F8	110.9 (4)
C38—C39—H39B	109.5	F6—B2—F8	109.7 (3)
H39A—C39—H39B	109.5	F7—B2—F5	110.4 (3)
C38—C39—H39C	109.5	F6—B2—F5	108.7 (3)
H39A—C39—H39C	109.5	F8—B2—F5	108.7 (3)
H39B—C39—H39C	109.5		
			
N1—C1—C2—C3	141 (17)	C13—C14—Fe2—C24	−50.2 (3)
N1—C1—C2—C6	−42 (17)	C18—C14—Fe2—C24	71.3 (2)
N1—C1—C2—Fe1	−130 (17)	C15—C14—Fe2—C24	−169.03 (18)
C1—C2—C3—C4	177.9 (3)	C13—C14—Fe2—C21	39.3 (3)
C6—C2—C3—C4	0.5 (3)	C18—C14—Fe2—C21	160.74 (19)
Fe1—C2—C3—C4	59.8 (2)	C15—C14—Fe2—C21	−79.6 (2)
C1—C2—C3—Fe1	118.1 (3)	C13—C14—Fe2—C17	−159.4 (3)
C6—C2—C3—Fe1	−59.32 (19)	C18—C14—Fe2—C17	−37.9 (2)
C2—C3—C4—C5	−0.5 (3)	C15—C14—Fe2—C17	81.8 (2)
Fe1—C3—C4—C5	58.5 (2)	C13—C14—Fe2—C23	−92.4 (5)
C2—C3—C4—Fe1	−59.03 (19)	C18—C14—Fe2—C23	29.1 (5)
C3—C4—C5—C6	0.4 (3)	C15—C14—Fe2—C23	148.7 (4)
Fe1—C4—C5—C6	59.1 (2)	C13—C14—Fe2—C22	79.1 (4)
C3—C4—C5—Fe1	−58.7 (2)	C18—C14—Fe2—C22	−159.5 (3)
C4—C5—C6—C2	−0.1 (3)	C15—C14—Fe2—C22	−39.8 (4)
Fe1—C5—C6—C2	59.15 (19)	C13—C14—Fe2—C15	118.9 (4)
C4—C5—C6—Fe1	−59.2 (2)	C18—C14—Fe2—C15	−119.7 (3)
C1—C2—C6—C5	−177.7 (3)	C13—C14—Fe2—C16	156.9 (3)
C3—C2—C6—C5	−0.3 (3)	C18—C14—Fe2—C16	−81.7 (2)
Fe1—C2—C6—C5	−59.65 (19)	C15—C14—Fe2—C16	38.0 (2)
C1—C2—C6—Fe1	−118.1 (3)	C17—C18—Fe2—C20	155.9 (2)
C3—C2—C6—Fe1	59.38 (19)	C14—C18—Fe2—C20	−85.6 (2)
N4—C7—C8—C12	29 (33)	C17—C18—Fe2—C14	−118.4 (3)
N4—C7—C8—C9	−157 (32)	C17—C18—Fe2—C24	113.2 (2)
N4—C7—C8—Fe1	116 (32)	C14—C18—Fe2—C24	−128.34 (19)
C7—C8—C9—C10	−175.9 (3)	C17—C18—Fe2—C21	−168.7 (3)
C12—C8—C9—C10	−1.1 (3)	C14—C18—Fe2—C21	−50.2 (4)
Fe1—C8—C9—C10	−60.8 (2)	C14—C18—Fe2—C17	118.4 (3)
C7—C8—C9—Fe1	−115.0 (3)	C17—C18—Fe2—C23	71.4 (2)
C12—C8—C9—Fe1	59.71 (19)	C14—C18—Fe2—C23	−170.15 (18)
C8—C9—C10—C11	0.9 (3)	C17—C18—Fe2—C22	38.7 (5)
Fe1—C9—C10—C11	−58.5 (2)	C14—C18—Fe2—C22	157.2 (3)
C8—C9—C10—Fe1	59.34 (19)	C17—C18—Fe2—C15	−80.6 (2)
C9—C10—C11—C12	−0.3 (3)	C14—C18—Fe2—C15	37.82 (19)
Fe1—C10—C11—C12	−58.2 (2)	C17—C18—Fe2—C16	−37.3 (2)
C9—C10—C11—Fe1	57.9 (2)	C14—C18—Fe2—C16	81.2 (2)
C10—C11—C12—C8	−0.4 (3)	C23—C24—Fe2—C20	118.1 (3)
Fe1—C11—C12—C8	−58.89 (19)	C23—C24—Fe2—C14	−162.39 (18)
C10—C11—C12—Fe1	58.5 (2)	C20—C24—Fe2—C14	79.5 (2)
C7—C8—C12—C11	175.8 (3)	C23—C24—Fe2—C18	−121.63 (19)
C9—C8—C12—C11	1.0 (3)	C20—C24—Fe2—C18	120.30 (18)
Fe1—C8—C12—C11	60.38 (19)	C23—C24—Fe2—C21	80.26 (19)
C7—C8—C12—Fe1	115.4 (3)	C20—C24—Fe2—C21	−37.80 (18)
C9—C8—C12—Fe1	−59.43 (19)	C23—C24—Fe2—C17	−79.2 (2)
N2—C13—C14—C18	113 (7)	C20—C24—Fe2—C17	162.76 (18)
N2—C13—C14—C15	−68 (7)	C20—C24—Fe2—C23	−118.1 (3)
N2—C13—C14—Fe2	−157 (7)	C23—C24—Fe2—C22	36.76 (18)
C13—C14—C15—C16	−179.9 (3)	C20—C24—Fe2—C22	−81.31 (19)
C18—C14—C15—C16	−0.6 (3)	C23—C24—Fe2—C15	165.1 (5)
Fe2—C14—C15—C16	−59.9 (2)	C20—C24—Fe2—C15	47.0 (6)
C13—C14—C15—Fe2	−120.0 (3)	C23—C24—Fe2—C16	−45.7 (4)
C18—C14—C15—Fe2	59.27 (19)	C20—C24—Fe2—C16	−163.8 (3)
C14—C15—C16—C17	0.5 (3)	C22—C21—Fe2—C20	−118.0 (3)
Fe2—C15—C16—C17	−58.4 (2)	C22—C21—Fe2—C14	157.19 (19)
C14—C15—C16—Fe2	58.96 (19)	C20—C21—Fe2—C14	−84.8 (2)
C15—C16—C17—C18	−0.3 (3)	C22—C21—Fe2—C18	−165.4 (3)
Fe2—C16—C17—C18	−58.9 (2)	C20—C21—Fe2—C18	−47.4 (4)
C15—C16—C17—Fe2	58.6 (2)	C22—C21—Fe2—C24	−80.3 (2)
C16—C17—C18—C14	−0.1 (3)	C20—C21—Fe2—C24	37.71 (17)
Fe2—C17—C18—C14	−59.29 (19)	C22—C21—Fe2—C17	36.1 (5)
C16—C17—C18—Fe2	59.2 (2)	C20—C21—Fe2—C17	154.1 (4)
C13—C14—C18—C17	179.7 (3)	C22—C21—Fe2—C23	−36.46 (19)
C15—C14—C18—C17	0.4 (3)	C20—C21—Fe2—C23	81.53 (19)
Fe2—C14—C18—C17	59.9 (2)	C20—C21—Fe2—C22	118.0 (3)
C13—C14—C18—Fe2	119.8 (3)	C22—C21—Fe2—C15	113.9 (2)
C15—C14—C18—Fe2	−59.50 (19)	C20—C21—Fe2—C15	−128.08 (18)
N5—C19—C20—C24	100 (7)	C22—C21—Fe2—C16	72.2 (2)
N5—C19—C20—C21	−72 (7)	C20—C21—Fe2—C16	−169.85 (18)
N5—C19—C20—Fe2	13 (7)	C18—C17—Fe2—C20	−55.6 (4)
C19—C20—C21—C22	173.5 (3)	C16—C17—Fe2—C20	−175.6 (3)
C24—C20—C21—C22	0.4 (3)	C18—C17—Fe2—C14	38.6 (2)
Fe2—C20—C21—C22	59.90 (18)	C16—C17—Fe2—C14	−81.4 (2)
C19—C20—C21—Fe2	113.6 (3)	C16—C17—Fe2—C18	−119.9 (3)
C24—C20—C21—Fe2	−59.47 (19)	C18—C17—Fe2—C24	−83.7 (2)
C20—C21—C22—C23	−0.3 (3)	C16—C17—Fe2—C24	156.37 (19)
Fe2—C21—C22—C23	58.6 (2)	C18—C17—Fe2—C21	168.3 (3)
C20—C21—C22—Fe2	−58.86 (18)	C16—C17—Fe2—C21	48.4 (5)
C21—C22—C23—C24	0.1 (3)	C18—C17—Fe2—C23	−125.2 (2)
Fe2—C22—C23—C24	58.4 (2)	C16—C17—Fe2—C23	114.8 (2)
C21—C22—C23—Fe2	−58.3 (2)	C18—C17—Fe2—C22	−164.8 (2)
C22—C23—C24—C20	0.2 (3)	C16—C17—Fe2—C22	75.3 (2)
Fe2—C23—C24—C20	59.03 (19)	C18—C17—Fe2—C15	83.2 (2)
C22—C23—C24—Fe2	−58.8 (2)	C16—C17—Fe2—C15	−36.71 (19)
C19—C20—C24—C23	−173.3 (3)	C18—C17—Fe2—C16	119.9 (3)
C21—C20—C24—C23	−0.4 (3)	C22—C23—Fe2—C20	81.53 (18)
Fe2—C20—C24—C23	−59.94 (19)	C24—C23—Fe2—C20	−38.82 (18)
C19—C20—C24—Fe2	−113.4 (3)	C22—C23—Fe2—C14	174.8 (4)
C21—C20—C24—Fe2	59.55 (19)	C24—C23—Fe2—C14	54.5 (5)
N3—C25—C26—C27	153 (7)	C22—C23—Fe2—C18	−162.68 (18)
N3—C25—C26—C30	−31 (7)	C24—C23—Fe2—C18	77.0 (2)
N3—C25—C26—Fe3	−119 (7)	C22—C23—Fe2—C24	120.3 (2)
C25—C26—C27—C28	177.3 (3)	C22—C23—Fe2—C21	36.77 (17)
C30—C26—C27—C28	0.6 (3)	C24—C23—Fe2—C21	−83.57 (19)
Fe3—C26—C27—C28	59.75 (19)	C22—C23—Fe2—C17	−123.27 (19)
C25—C26—C27—Fe3	117.6 (3)	C24—C23—Fe2—C17	116.4 (2)
C30—C26—C27—Fe3	−59.15 (18)	C24—C23—Fe2—C22	−120.3 (2)
C26—C27—C28—C29	−0.4 (3)	C22—C23—Fe2—C15	−52.3 (3)
Fe3—C27—C28—C29	58.43 (19)	C24—C23—Fe2—C15	−172.6 (3)
C26—C27—C28—Fe3	−58.82 (19)	C22—C23—Fe2—C16	−81.9 (2)
C27—C28—C29—C30	0.0 (3)	C24—C23—Fe2—C16	157.75 (19)
Fe3—C28—C29—C30	58.5 (2)	C23—C22—Fe2—C20	−82.00 (19)
C27—C28—C29—Fe3	−58.43 (19)	C21—C22—Fe2—C20	39.00 (18)
C28—C29—C30—C26	0.3 (3)	C23—C22—Fe2—C14	−176.1 (3)
Fe3—C29—C30—C26	59.11 (18)	C21—C22—Fe2—C14	−55.1 (4)
C28—C29—C30—Fe3	−58.8 (2)	C23—C22—Fe2—C18	44.3 (4)
C25—C26—C30—C29	−177.3 (3)	C21—C22—Fe2—C18	165.3 (3)
C27—C26—C30—C29	−0.6 (3)	C23—C22—Fe2—C24	−37.33 (18)
Fe3—C26—C30—C29	−60.12 (19)	C21—C22—Fe2—C24	83.67 (19)
C25—C26—C30—Fe3	−117.2 (3)	C23—C22—Fe2—C21	−121.0 (3)
C27—C26—C30—Fe3	59.53 (19)	C23—C22—Fe2—C17	73.0 (2)
N6—C31—C32—C33	−109 (33)	C21—C22—Fe2—C17	−166.0 (2)
N6—C31—C32—C36	77 (33)	C21—C22—Fe2—C23	121.0 (3)
N6—C31—C32—Fe3	164 (100)	C23—C22—Fe2—C15	154.83 (18)
C31—C32—C33—C34	−176.4 (3)	C21—C22—Fe2—C15	−84.2 (2)
C36—C32—C33—C34	−0.8 (3)	C23—C22—Fe2—C16	113.6 (2)
Fe3—C32—C33—C34	−60.14 (19)	C21—C22—Fe2—C16	−125.4 (2)
C31—C32—C33—Fe3	−116.3 (3)	C16—C15—Fe2—C20	−163.7 (2)
C36—C32—C33—Fe3	59.3 (2)	C14—C15—Fe2—C20	78.3 (2)
C32—C33—C34—C35	0.8 (3)	C16—C15—Fe2—C14	118.0 (3)
Fe3—C33—C34—C35	−58.2 (2)	C16—C15—Fe2—C18	80.4 (2)
C32—C33—C34—Fe3	59.03 (19)	C14—C15—Fe2—C18	−37.66 (18)
C33—C34—C35—C36	−0.5 (3)	C16—C15—Fe2—C24	158.1 (5)
Fe3—C34—C35—C36	−58.9 (2)	C14—C15—Fe2—C24	40.1 (6)
C33—C34—C35—Fe3	58.4 (2)	C16—C15—Fe2—C21	−121.8 (2)
C34—C35—C36—C32	0.0 (3)	C14—C15—Fe2—C21	120.20 (18)
Fe3—C35—C36—C32	−59.24 (19)	C16—C15—Fe2—C17	36.9 (2)
C34—C35—C36—Fe3	59.2 (2)	C14—C15—Fe2—C17	−81.1 (2)
C31—C32—C36—C35	176.1 (3)	C16—C15—Fe2—C23	−43.0 (4)
C33—C32—C36—C35	0.5 (3)	C14—C15—Fe2—C23	−161.0 (2)
Fe3—C32—C36—C35	60.0 (2)	C16—C15—Fe2—C22	−78.8 (2)
C31—C32—C36—Fe3	116.0 (3)	C14—C15—Fe2—C22	163.18 (17)
C33—C32—C36—Fe3	−59.52 (19)	C14—C15—Fe2—C16	−118.0 (3)
C2—C1—N1—Cu1	81 (18)	C15—C16—Fe2—C20	52.6 (5)
C14—C13—N2—Cu1	21 (7)	C17—C16—Fe2—C20	173.3 (4)
C26—C25—N3—Cu1	135 (6)	C15—C16—Fe2—C14	−38.96 (19)
C8—C7—N4—Cu2	95 (33)	C17—C16—Fe2—C14	81.8 (2)
C20—C19—N5—Cu2	16 (9)	C15—C16—Fe2—C18	−83.5 (2)
C32—C31—N6—Cu2	54 (34)	C17—C16—Fe2—C18	37.24 (19)
C7—C8—Fe1—C2	5.3 (3)	C15—C16—Fe2—C24	−169.4 (3)
C12—C8—Fe1—C2	124.68 (18)	C17—C16—Fe2—C24	−48.6 (4)
C9—C8—Fe1—C2	−115.53 (19)	C15—C16—Fe2—C21	77.6 (2)
C7—C8—Fe1—C9	120.8 (3)	C17—C16—Fe2—C21	−161.72 (18)
C12—C8—Fe1—C9	−119.8 (3)	C15—C16—Fe2—C17	−120.7 (3)
C7—C8—Fe1—C5	−79.6 (4)	C15—C16—Fe2—C23	159.01 (19)
C12—C8—Fe1—C5	39.8 (4)	C17—C16—Fe2—C23	−80.3 (2)
C9—C8—Fe1—C5	159.6 (3)	C15—C16—Fe2—C22	117.5 (2)
C7—C8—Fe1—C12	−119.4 (3)	C17—C16—Fe2—C22	−121.8 (2)
C9—C8—Fe1—C12	119.8 (3)	C17—C16—Fe2—C15	120.7 (3)
C7—C8—Fe1—C6	−39.7 (3)	C31—C32—Fe3—C26	−4.0 (3)
C12—C8—Fe1—C6	79.7 (2)	C33—C32—Fe3—C26	−123.73 (18)
C9—C8—Fe1—C6	−160.49 (19)	C36—C32—Fe3—C26	116.59 (18)
C7—C8—Fe1—C3	49.7 (3)	C31—C32—Fe3—C30	−48.7 (3)
C12—C8—Fe1—C3	169.10 (17)	C33—C32—Fe3—C30	−168.44 (17)
C9—C8—Fe1—C3	−71.1 (2)	C36—C32—Fe3—C30	71.9 (2)
C7—C8—Fe1—C4	90.2 (4)	C31—C32—Fe3—C36	−120.6 (3)
C12—C8—Fe1—C4	−150.4 (4)	C33—C32—Fe3—C36	119.7 (2)
C9—C8—Fe1—C4	−30.6 (5)	C31—C32—Fe3—C33	119.7 (3)
C7—C8—Fe1—C10	158.9 (3)	C36—C32—Fe3—C33	−119.7 (2)
C12—C8—Fe1—C10	−81.70 (19)	C31—C32—Fe3—C35	−158.8 (3)
C9—C8—Fe1—C10	38.10 (19)	C33—C32—Fe3—C35	81.53 (19)
C7—C8—Fe1—C11	−157.5 (3)	C36—C32—Fe3—C35	−38.15 (18)
C12—C8—Fe1—C11	−38.10 (18)	C31—C32—Fe3—C27	40.7 (3)
C9—C8—Fe1—C11	81.7 (2)	C33—C32—Fe3—C27	−79.0 (2)
C1—C2—Fe1—C8	1.4 (3)	C36—C32—Fe3—C27	161.30 (17)
C3—C2—Fe1—C8	121.77 (18)	C31—C32—Fe3—C29	−89.5 (4)
C6—C2—Fe1—C8	−118.40 (18)	C33—C32—Fe3—C29	150.8 (3)
C1—C2—Fe1—C9	−44.1 (3)	C36—C32—Fe3—C29	31.1 (4)
C3—C2—Fe1—C9	76.3 (2)	C31—C32—Fe3—C28	80.4 (4)
C6—C2—Fe1—C9	−163.86 (17)	C33—C32—Fe3—C28	−39.3 (4)
C1—C2—Fe1—C5	158.1 (3)	C36—C32—Fe3—C28	−159.0 (3)
C3—C2—Fe1—C5	−81.50 (19)	C31—C32—Fe3—C34	157.2 (3)
C6—C2—Fe1—C5	38.34 (18)	C33—C32—Fe3—C34	37.54 (18)
C1—C2—Fe1—C12	45.6 (3)	C36—C32—Fe3—C34	−82.15 (19)
C3—C2—Fe1—C12	165.98 (18)	C25—C26—Fe3—C32	−2.4 (3)
C6—C2—Fe1—C12	−74.2 (2)	C27—C26—Fe3—C32	118.05 (18)
C1—C2—Fe1—C6	119.8 (3)	C30—C26—Fe3—C32	−122.34 (19)
C3—C2—Fe1—C6	−119.8 (2)	C25—C26—Fe3—C30	120.0 (3)
C1—C2—Fe1—C3	−120.4 (3)	C27—C26—Fe3—C30	−119.6 (3)
C6—C2—Fe1—C3	119.8 (2)	C25—C26—Fe3—C36	42.6 (3)
C1—C2—Fe1—C4	−158.3 (3)	C27—C26—Fe3—C36	163.04 (18)
C3—C2—Fe1—C4	−37.89 (18)	C30—C26—Fe3—C36	−77.4 (2)
C6—C2—Fe1—C4	81.95 (19)	C25—C26—Fe3—C33	−46.2 (3)
C1—C2—Fe1—C10	−82.5 (4)	C27—C26—Fe3—C33	74.2 (2)
C3—C2—Fe1—C10	37.9 (4)	C30—C26—Fe3—C33	−166.19 (18)
C6—C2—Fe1—C10	157.8 (3)	C25—C26—Fe3—C35	81.7 (4)
C1—C2—Fe1—C11	86.8 (5)	C27—C26—Fe3—C35	−157.9 (3)
C3—C2—Fe1—C11	−152.8 (4)	C30—C26—Fe3—C35	−38.3 (4)
C6—C2—Fe1—C11	−33.0 (5)	C25—C26—Fe3—C27	−120.4 (3)
C10—C9—Fe1—C8	117.5 (3)	C30—C26—Fe3—C27	119.6 (3)
C10—C9—Fe1—C2	−156.48 (18)	C25—C26—Fe3—C29	157.7 (3)
C8—C9—Fe1—C2	86.1 (2)	C27—C26—Fe3—C29	−81.85 (19)
C10—C9—Fe1—C5	−43.0 (4)	C30—C26—Fe3—C29	37.75 (18)
C8—C9—Fe1—C5	−160.4 (3)	C25—C26—Fe3—C28	−158.4 (3)
C10—C9—Fe1—C12	79.7 (2)	C27—C26—Fe3—C28	−37.95 (17)
C8—C9—Fe1—C12	−37.71 (18)	C30—C26—Fe3—C28	81.65 (19)
C10—C9—Fe1—C6	171.0 (3)	C25—C26—Fe3—C34	−85.9 (5)
C8—C9—Fe1—C6	53.5 (4)	C27—C26—Fe3—C34	34.6 (5)
C10—C9—Fe1—C3	−114.64 (19)	C30—C26—Fe3—C34	154.2 (4)
C8—C9—Fe1—C3	127.91 (19)	C29—C30—Fe3—C32	−161.29 (17)
C10—C9—Fe1—C4	−73.7 (2)	C26—C30—Fe3—C32	80.4 (2)
C8—C9—Fe1—C4	168.84 (18)	C29—C30—Fe3—C26	118.3 (3)
C8—C9—Fe1—C10	−117.5 (3)	C29—C30—Fe3—C36	−120.22 (18)
C10—C9—Fe1—C11	36.48 (19)	C26—C30—Fe3—C36	121.44 (19)
C8—C9—Fe1—C11	−81.0 (2)	C29—C30—Fe3—C33	167.0 (4)
C4—C5—Fe1—C8	174.9 (3)	C26—C30—Fe3—C33	48.7 (6)
C6—C5—Fe1—C8	54.3 (4)	C29—C30—Fe3—C35	−78.0 (2)
C4—C5—Fe1—C2	81.4 (2)	C26—C30—Fe3—C35	163.69 (18)
C6—C5—Fe1—C2	−39.14 (19)	C29—C30—Fe3—C27	80.63 (18)
C4—C5—Fe1—C9	−42.4 (4)	C26—C30—Fe3—C27	−37.70 (18)
C6—C5—Fe1—C9	−162.9 (3)	C26—C30—Fe3—C29	−118.3 (3)
C4—C5—Fe1—C12	−155.58 (19)	C29—C30—Fe3—C28	37.16 (17)
C6—C5—Fe1—C12	83.9 (2)	C26—C30—Fe3—C28	−81.17 (19)
C4—C5—Fe1—C6	120.6 (3)	C29—C30—Fe3—C34	−44.9 (3)
C4—C5—Fe1—C3	36.79 (19)	C26—C30—Fe3—C34	−163.3 (3)
C6—C5—Fe1—C3	−83.8 (2)	C35—C36—Fe3—C32	−117.9 (3)
C6—C5—Fe1—C4	−120.6 (3)	C35—C36—Fe3—C26	157.23 (19)
C4—C5—Fe1—C10	−73.5 (2)	C32—C36—Fe3—C26	−84.8 (2)
C6—C5—Fe1—C10	165.97 (19)	C35—C36—Fe3—C30	114.5 (2)
C4—C5—Fe1—C11	−114.4 (2)	C32—C36—Fe3—C30	−127.53 (17)
C6—C5—Fe1—C11	125.09 (19)	C35—C36—Fe3—C33	−80.3 (2)
C11—C12—Fe1—C8	−117.8 (3)	C32—C36—Fe3—C33	37.63 (17)
C11—C12—Fe1—C2	163.72 (18)	C32—C36—Fe3—C35	117.9 (3)
C8—C12—Fe1—C2	−78.5 (2)	C35—C36—Fe3—C27	−168.6 (3)
C11—C12—Fe1—C9	−79.9 (2)	C32—C36—Fe3—C27	−50.7 (4)
C8—C12—Fe1—C9	37.88 (18)	C35—C36—Fe3—C29	73.2 (2)
C11—C12—Fe1—C5	78.1 (2)	C32—C36—Fe3—C29	−168.83 (17)
C8—C12—Fe1—C5	−164.09 (18)	C35—C36—Fe3—C28	40.6 (4)
C11—C12—Fe1—C6	121.33 (19)	C32—C36—Fe3—C28	158.5 (3)
C8—C12—Fe1—C6	−120.88 (18)	C35—C36—Fe3—C34	−37.4 (2)
C11—C12—Fe1—C3	−155.8 (5)	C32—C36—Fe3—C34	80.55 (19)
C8—C12—Fe1—C3	−38.0 (6)	C34—C33—Fe3—C32	118.5 (3)
C11—C12—Fe1—C4	41.2 (4)	C34—C33—Fe3—C26	−162.69 (19)
C8—C12—Fe1—C4	159.0 (3)	C32—C33—Fe3—C26	78.8 (2)
C11—C12—Fe1—C10	−36.83 (19)	C34—C33—Fe3—C30	158.1 (4)
C8—C12—Fe1—C10	80.95 (19)	C32—C33—Fe3—C30	39.6 (6)
C8—C12—Fe1—C11	117.8 (3)	C34—C33—Fe3—C36	80.6 (2)
C5—C6—Fe1—C8	−158.71 (19)	C32—C33—Fe3—C36	−37.86 (17)
C2—C6—Fe1—C8	83.6 (2)	C34—C33—Fe3—C35	37.1 (2)
C5—C6—Fe1—C2	117.7 (3)	C32—C33—Fe3—C35	−81.41 (19)
C5—C6—Fe1—C9	160.8 (3)	C34—C33—Fe3—C27	−120.6 (2)
C2—C6—Fe1—C9	43.2 (4)	C32—C33—Fe3—C27	120.92 (18)
C2—C6—Fe1—C5	−117.7 (3)	C34—C33—Fe3—C29	−41.0 (4)
C5—C6—Fe1—C12	−115.1 (2)	C32—C33—Fe3—C29	−159.5 (2)
C2—C6—Fe1—C12	127.28 (18)	C34—C33—Fe3—C28	−77.7 (2)
C5—C6—Fe1—C3	80.0 (2)	C32—C33—Fe3—C28	163.85 (16)
C2—C6—Fe1—C3	−37.62 (17)	C32—C33—Fe3—C34	−118.5 (3)
C5—C6—Fe1—C4	36.8 (2)	C36—C35—Fe3—C32	39.01 (19)
C2—C6—Fe1—C4	−80.86 (19)	C34—C35—Fe3—C32	−80.9 (2)
C5—C6—Fe1—C10	−35.9 (4)	C36—C35—Fe3—C26	−54.4 (4)
C2—C6—Fe1—C10	−153.5 (3)	C34—C35—Fe3—C26	−174.3 (3)
C5—C6—Fe1—C11	−72.9 (2)	C36—C35—Fe3—C30	−82.3 (2)
C2—C6—Fe1—C11	169.48 (17)	C34—C35—Fe3—C30	157.73 (19)
C4—C3—Fe1—C8	161.02 (18)	C34—C35—Fe3—C36	−119.9 (3)
C2—C3—Fe1—C8	−80.8 (2)	C36—C35—Fe3—C33	83.5 (2)
C4—C3—Fe1—C2	−118.2 (2)	C34—C35—Fe3—C33	−36.45 (19)
C4—C3—Fe1—C9	120.14 (18)	C36—C35—Fe3—C27	169.2 (3)
C2—C3—Fe1—C9	−121.69 (18)	C34—C35—Fe3—C27	49.2 (4)
C4—C3—Fe1—C5	−36.85 (18)	C36—C35—Fe3—C29	−123.1 (2)
C2—C3—Fe1—C5	81.33 (19)	C34—C35—Fe3—C29	116.9 (2)
C4—C3—Fe1—C12	−168.4 (4)	C36—C35—Fe3—C28	−163.49 (18)
C2—C3—Fe1—C12	−50.2 (5)	C34—C35—Fe3—C28	76.6 (2)
C4—C3—Fe1—C6	−80.43 (19)	C36—C35—Fe3—C34	119.9 (3)
C2—C3—Fe1—C6	37.74 (17)	C28—C27—Fe3—C32	158.05 (17)
C2—C3—Fe1—C4	118.2 (2)	C26—C27—Fe3—C32	−83.6 (2)
C4—C3—Fe1—C10	78.6 (2)	C28—C27—Fe3—C26	−118.4 (2)
C2—C3—Fe1—C10	−163.25 (17)	C28—C27—Fe3—C30	−80.30 (19)
C4—C3—Fe1—C11	46.2 (3)	C26—C27—Fe3—C30	38.06 (18)
C2—C3—Fe1—C11	164.4 (2)	C28—C27—Fe3—C36	−163.8 (3)
C5—C4—Fe1—C8	−174.2 (3)	C26—C27—Fe3—C36	−45.4 (4)
C3—C4—Fe1—C8	−53.6 (4)	C28—C27—Fe3—C33	115.04 (18)
C5—C4—Fe1—C2	−81.8 (2)	C26—C27—Fe3—C33	−126.60 (18)
C3—C4—Fe1—C2	38.83 (17)	C28—C27—Fe3—C35	37.1 (4)
C5—C4—Fe1—C9	162.41 (19)	C26—C27—Fe3—C35	155.4 (3)
C3—C4—Fe1—C9	−77.0 (2)	C28—C27—Fe3—C29	−37.05 (17)
C3—C4—Fe1—C5	120.6 (3)	C26—C27—Fe3—C29	81.31 (18)
C5—C4—Fe1—C12	53.0 (3)	C26—C27—Fe3—C28	118.4 (2)
C3—C4—Fe1—C12	173.6 (2)	C28—C27—Fe3—C34	73.4 (2)
C5—C4—Fe1—C6	−37.00 (19)	C26—C27—Fe3—C34	−168.21 (18)
C3—C4—Fe1—C6	83.59 (19)	C30—C29—Fe3—C32	53.8 (4)
C5—C4—Fe1—C3	−120.6 (3)	C28—C29—Fe3—C32	174.2 (3)
C5—C4—Fe1—C10	122.7 (2)	C30—C29—Fe3—C26	−39.00 (17)
C3—C4—Fe1—C10	−116.68 (19)	C28—C29—Fe3—C26	81.42 (18)
C5—C4—Fe1—C11	81.8 (2)	C28—C29—Fe3—C30	120.4 (2)
C3—C4—Fe1—C11	−157.58 (18)	C30—C29—Fe3—C36	77.5 (2)
C9—C10—Fe1—C8	−39.52 (19)	C28—C29—Fe3—C36	−162.05 (17)
C11—C10—Fe1—C8	81.74 (19)	C30—C29—Fe3—C33	−172.7 (2)
C9—C10—Fe1—C2	54.2 (4)	C28—C29—Fe3—C33	−52.3 (3)
C11—C10—Fe1—C2	175.4 (3)	C30—C29—Fe3—C35	117.47 (18)
C11—C10—Fe1—C9	121.3 (3)	C28—C29—Fe3—C35	−122.11 (18)
C9—C10—Fe1—C5	162.14 (19)	C30—C29—Fe3—C27	−83.52 (18)
C11—C10—Fe1—C5	−76.6 (2)	C28—C29—Fe3—C27	36.89 (17)
C9—C10—Fe1—C12	−84.4 (2)	C30—C29—Fe3—C28	−120.4 (2)
C11—C10—Fe1—C12	36.87 (18)	C30—C29—Fe3—C34	158.72 (18)
C9—C10—Fe1—C6	−171.2 (3)	C28—C29—Fe3—C34	−80.86 (19)
C11—C10—Fe1—C6	−50.0 (4)	C27—C28—Fe3—C32	−54.6 (4)
C9—C10—Fe1—C3	81.5 (2)	C29—C28—Fe3—C32	−175.1 (3)
C11—C10—Fe1—C3	−157.24 (18)	C27—C28—Fe3—C26	38.68 (17)
C9—C10—Fe1—C4	122.3 (2)	C29—C28—Fe3—C26	−81.86 (18)
C11—C10—Fe1—C4	−116.41 (19)	C27—C28—Fe3—C30	83.69 (18)
C9—C10—Fe1—C11	−121.3 (3)	C29—C28—Fe3—C30	−36.85 (17)
C12—C11—Fe1—C8	39.15 (18)	C27—C28—Fe3—C36	165.1 (3)
C10—C11—Fe1—C8	−81.39 (19)	C29—C28—Fe3—C36	44.5 (4)
C12—C11—Fe1—C2	−52.6 (5)	C27—C28—Fe3—C33	−83.4 (2)
C10—C11—Fe1—C2	−173.1 (4)	C29—C28—Fe3—C33	156.05 (17)
C12—C11—Fe1—C9	84.2 (2)	C27—C28—Fe3—C35	−165.22 (18)
C10—C11—Fe1—C9	−36.35 (18)	C29—C28—Fe3—C35	74.2 (2)
C12—C11—Fe1—C5	−118.85 (19)	C29—C28—Fe3—C27	−120.5 (2)
C10—C11—Fe1—C5	120.61 (19)	C27—C28—Fe3—C29	120.5 (2)
C10—C11—Fe1—C12	−120.5 (3)	C27—C28—Fe3—C34	−124.06 (19)
C12—C11—Fe1—C6	−78.6 (2)	C29—C28—Fe3—C34	115.40 (18)
C10—C11—Fe1—C6	160.89 (18)	C33—C34—Fe3—C32	−38.65 (19)
C12—C11—Fe1—C3	167.5 (2)	C35—C34—Fe3—C32	82.0 (2)
C10—C11—Fe1—C3	47.0 (3)	C33—C34—Fe3—C26	51.7 (5)
C12—C11—Fe1—C4	−160.64 (18)	C35—C34—Fe3—C26	172.4 (4)
C10—C11—Fe1—C4	78.8 (2)	C33—C34—Fe3—C30	−168.1 (3)
C12—C11—Fe1—C10	120.5 (3)	C35—C34—Fe3—C30	−47.4 (4)
C19—C20—Fe2—C14	−2.4 (3)	C33—C34—Fe3—C36	−83.5 (2)
C24—C20—Fe2—C14	−123.31 (19)	C35—C34—Fe3—C36	37.23 (19)
C21—C20—Fe2—C14	116.92 (18)	C35—C34—Fe3—C33	120.7 (3)
C19—C20—Fe2—C18	42.5 (3)	C33—C34—Fe3—C35	−120.7 (3)
C24—C20—Fe2—C18	−78.5 (2)	C33—C34—Fe3—C27	78.5 (2)
C21—C20—Fe2—C18	161.77 (18)	C35—C34—Fe3—C27	−160.82 (19)
C19—C20—Fe2—C24	120.9 (3)	C33—C34—Fe3—C29	160.70 (18)
C21—C20—Fe2—C24	−119.8 (2)	C35—C34—Fe3—C29	−78.6 (2)
C19—C20—Fe2—C21	−119.3 (3)	C33—C34—Fe3—C28	118.76 (19)
C24—C20—Fe2—C21	119.8 (2)	C35—C34—Fe3—C28	−120.54 (19)
C19—C20—Fe2—C17	82.0 (4)	C1—N1—Cu1—N3	136 (5)
C24—C20—Fe2—C17	−38.9 (4)	C1—N1—Cu1—N2	−23 (5)
C21—C20—Fe2—C17	−158.7 (3)	C1—N1—Cu1—O1	−122 (5)
C19—C20—Fe2—C23	159.2 (3)	C25—N3—Cu1—N1	−95 (6)
C24—C20—Fe2—C23	38.30 (19)	C25—N3—Cu1—N2	65 (6)
C21—C20—Fe2—C23	−81.47 (19)	C25—N3—Cu1—O1	165 (6)
C19—C20—Fe2—C22	−157.4 (3)	C13—N2—Cu1—N1	−128.5 (8)
C24—C20—Fe2—C22	81.6 (2)	C13—N2—Cu1—N3	69.4 (8)
C21—C20—Fe2—C22	−38.12 (18)	C13—N2—Cu1—O1	−31.9 (8)
C19—C20—Fe2—C15	−46.4 (3)	C7—N4—Cu2—N5	−112.1 (15)
C24—C20—Fe2—C15	−167.36 (18)	C7—N4—Cu2—N6	61.3 (16)
C21—C20—Fe2—C15	72.9 (2)	C19—N5—Cu2—N4	−111.5 (15)
C19—C20—Fe2—C16	−87.6 (5)	C19—N5—Cu2—N6	74.9 (15)
C24—C20—Fe2—C16	151.5 (4)	C31—N6—Cu2—N4	−123 (2)
C21—C20—Fe2—C16	31.7 (5)	C31—N6—Cu2—N5	51 (2)
C13—C14—Fe2—C20	−5.9 (3)	C39—C38—O1—Cu1	−13.3 (4)
C18—C14—Fe2—C20	115.6 (2)	C37—C38—O1—Cu1	166.6 (2)
C15—C14—Fe2—C20	−124.72 (19)	N1—Cu1—O1—C38	−175.9 (3)
C13—C14—Fe2—C18	−121.5 (4)	N3—Cu1—O1—C38	−48.9 (3)
C15—C14—Fe2—C18	119.7 (3)	N2—Cu1—O1—C38	67.3 (3)

## References

[bb1] Altmannshofer, S., Herdtweck, E., Köhler, F. H., Miller, R., Mölle, R., Scheidt, E.-W., Scherer, W. & Train, C. (2008). *Chem. Eur. J.* **14**, 8013–8024. 10.1002/chem.200701538 18645991

[bb2] Benmansour, S., Setifi, F., Triki, S., Thetiot, F., Sala-Pala, J., Gomez-Garcia, C.-J. & Colacio, E. (2009). *Polyhedron*, pp. 1308–1314.

[bb3] Bondi, A. (1964). *J. Phys. Chem.* **68**, 441–451.

[bb4] Bonniard, L., Kahlal, S., Diallo, A. K., Ornelas, C., Roisnel, T., Manca, G., Rodrigues, J., Ruiz, J., Astruc, D. & Saillard, J. Y. (2011). *Inorg. Chem.* **50**, 114–124. 10.1021/ic101415c 21117620

[bb5] Bowmaker, G. A., Gill, D. S., Skelton, B. W., Somers, N. & White, A. H. (2004). *Z. Naturforsch. Teil B*, pp. 1307–1313.

[bb6] Burgun, A., Gendron, F., Schauer, P. A., Skelton, B. W., Low, P. J., Costuas, K., Halet, J.-F., Bruce, M. I. & Lapinte, C. (2013). *Organometallics*, **32**, 5015–5025.

[bb7] Buschbeck, R., Low, P. J. & Lang, H. (2011). *Coord. Chem. Rev.* **255**, 241–272.

[bb8] Chesnut, D. J., Kusnetzow, A. & Zubieta, J. (1998). *J. Chem. Soc. Dalton Trans.* pp. 4081–4084.

[bb10] Díez, A., Fernández, J., Lalinde, E., Moreno, M. T. & Sánchez, S. (2008). *Dalton Trans.* pp. 4926–4936. 10.1039/b806572a 18766225

[bb9] Díez, Á., Lalinde, E., Moreno, M. T. & Sánchez, S. (2009). *Dalton Trans.* pp. 3434–3446. 10.1039/b822171e 19381406

[bb11] Dong, F.-Y., Li, Y.-T., Wu, Z.-Y., Sun, Y.-M., Sun, W., Liu, Z.-Q. & Song, Q.-L. (2008). *J. Inorg. Organomet. Polym.* **18**, 398–406.

[bb12] Dowling, N., Henry, P. M., Lewis, N. A. & Taube, H. (1981). *Inorg. Chem.* **20**, 2345–2348.

[bb13] Farrugia, L. J. (2012). *J. Appl. Cryst.* **45**, 849–854.

[bb14] Ferrara, J. D., Tessier-Youngs, C. & Youngs, W. J. (1987). *Organometallics*, **6**, 676–678.

[bb15] Frosch, W., Back, S., Müller, H., Köhler, K., Driess, A., Schiemenz, B., Huttner, G. & Lang, H. (2001). *J. Organomet. Chem.* **619**, 99–109.

[bb16] Frosch, W., Back, S., Rheinwald, G., Köhler, K., Pritzkow, H. & Lang, H. (2000). *Organometallics*, **19**, 4016–4024.

[bb17] Fu, D.-W., Ye, H.-Y., Ye, Q., Pan, K.-J. & Xiong, R.-G. (2008). *Dalton Trans.* pp. 874–877. 10.1039/b714293e 18259619

[bb18] Helten, H., Beckmann, M., Schnakenburg, G. & Streubel, R. (2010). *Eur. J. Inorg. Chem.* pp. 2337–2341.

[bb19] Jakob, A., Ecorchard, P., Linseis, M., Winter, R. F. & Lang, H. (2009). *J. Organomet. Chem.* **694**, 655–666.

[bb20] Janssen, M. D., Herres, M., Zsolnai, L., Grove, D. M., Spek, A. L., Lang, H. & van Koten, G. (1995). *Organometallics*, **14**, 1098–1100.

[bb21] Köhler, K., Pritzkow, H. & Lang, H. (1998). *J. Organomet. Chem.* **553**, 31–38.

[bb22] Lang, H., George, D. S. A. & Rheinwald, G. (2000). *Coord. Chem. Rev.* **206–207**, 101–197.

[bb23] Lang, H., Köhler, K. & Blau, S. (1995). *Coord. Chem. Rev.* **143**, 113–168.

[bb24] Lang, H., Packheiser, R. & Walfort, B. (2006). *Organometallics*, **25**, 1836–1850.

[bb25] McArdle, P. (1995). *J. Appl. Cryst.* **28**, 65. 10.1107/S1600576721008529 PMC8493623 34667454

[bb26] Osella, D., Gobetto, R., Nervi, C., Ravera, M., D’Amato, R. & Russo, M. V. (1998). *Inorg. Chem. Commun.* **1**, 239–245.

[bb27] Osgerby, J. & Pauson, P. (1961). *J. Chem. Soc.* pp. 4604–4609.

[bb28] Oxford Diffraction (2006). *CrysAlis CCD* and *CrysAlis RED*. Oxford Diffraction, Abingdon, England.

[bb29] Packheiser, R., Lohan, M., Bräuer, B., Justaud, F., Lapinte, C. & Lang, H. (2008). *J. Organomet. Chem.* **693**, 2898–2902.

[bb30] Sheldrick, G. M. (2008). *Acta Cryst.* A**64**, 112–122. 10.1107/S0108767307043930 18156677

[bb31] Sheldrick, G. M. (2015). *Acta Cryst.* C**71**, 3–8.

[bb32] Spek, A. L. (2007). Private communication (refcode YOSTOP01). CCDC, Cambridge, England.

[bb33] Strehler, F., Hildebrandt, H., Korb, M. & Lang, H. (2013). *Z. Anorg. Allg. Chem.* **639**, 1214–1219.

[bb34] Strehler, F., Hildebrandt, H., Korb, M., Rüffer, T. & Lang, H. (2014). *Organometallics*, **33**, 4279–4289.

[bb35] Vives, G., Carella, A., Sistach, S., Launay, J.-P. & Rapenne, G. (2006). *New J. Chem.* **30**, 1429–1438.

[bb36] Westrip, S. P. (2010). *J. Appl. Cryst.* **43**, 920–925.

